# FaTRAB1, a bZIP transcription factor, enhances anthocyanin biosynthesis in strawberry leaves via tissue-specific regulation

**DOI:** 10.1371/journal.pgen.1011888

**Published:** 2025-09-24

**Authors:** Min Yang, Chenghui Song, Yuanxiu Lin, Yunting Zhang, Mengyao Li, Qing Chen, Yong Zhang, Haoru Tang, Ya Luo

**Affiliations:** College of Horticulture, Sichuan Agricultural University, Chengdu, China; University of Kentucky, UNITED STATES OF AMERICA

## Abstract

The bZIP transcription factor FaTRAB1 has been extensively studied as a central regulator of plant adaptation to environmental stresses. However, its role in modulating anthocyanin biosynthesis remains unclear. This study reveals a novel function of FaTRAB1 in regulating anthocyanin accumulation in both strawberry leaves (*Fragaria* vesca ‘Ruegen’) and fruits (*Fragaria* × *ananassa* ‘Benihoppe’). FaTRAB1 is a nucleocytoplasmic-localized protein, exhibiting relatively higher expression in leaves and small green fruits. Stable overexpression of *FaTRAB1* in ‘Ruegen’ strawberry significantly enhanced anthocyanin levels in young, mature, and old leaves. Mass spectrometry and HPLC analysis identified three major anthocyanins in mature strawberry leaves: cyanidin-3-*O*-glucoside (C3G) and cyanidin-3,5-*O*-diglucoside (C3,5dG) (78.91%), and peonidin-3,5-*O*-diglucoside (Pg3,5dG, 12.6%), with pelargonidin-3-*O*-glucoside (Pg3G, 8.49%) as a minor component. Protein interaction assays demonstrated that FaTRAB1 likely competes with FabHLH3 for binding to FaMYB10 and FaTTG1, forming a novel regulatory complex that coordinately activates the promoter of anthocyanin structural genes (*FaF3’H*, *FaANS*, *FaUFG*T, and *FaOMT*). This mechanism preferentially promotes the accumulation of cyanidin derivatives (C3,5dG and C3G) in leaves, resulting in reddish-purple pigmentation, while Pg3,5dG and trace Pg3G contribute secondarily. In contrast, strawberry fruits predominantly accumulate Pg3G. Intriguingly, FaTRAB1 may slightly suppress FaMYB10/FaTTG1-mediated biosynthesis of Pg3G and cyanidin-based anthocyanins in fruits. Instead, it primarily enhances C3G accumulation through synergistic interaction with *FaF3’H*, leading to distinct pigmentation patterns between leaves and fruits. These findings elucidate the tissue-specific regulatory role of FaTRAB1 in strawberry anthocyanin accumulation, providing critical insights into organ-specific coloration mechanisms. This work establishes a theoretical foundation for improving strawberry color quality through targeted genetic manipulation.

## Author summary

Over the decades, the vibrant color of strawberry fruits has consistently captured the attention and interest of scientists. While the genetic mechanisms underlying strawberry fruit coloration have been preliminarily elucidated, whether anthocyanins can accumulate in strawberry leaves and the regulatory mechanisms involved remain poorly understood. In this study, we found that FaTRAB1 primarily promotes the accumulation of cyanidin derivatives in both strawberry leaves and fruits. Overexpressing FaTRAB1, the major pigments identified were cyanidin-3-*O*-glucoside (C3G) and cyanidin-3,5-*O*-diglucoside (C3,5dG), followed by peonidin-3,5-*O*-diglucoside (Pg3,5dG), and a small amount of pelargonidin-3-*O*-glucoside (Pg3G), resulting in the formation of purplish-red strawberry leaves. The key molecular mechanism involves FaTRAB1 activating the expression of *F3’H*, *ANS*, *UFGT*, and *OMT*, with *F3’H* being particularly critical for FaTRAB1-mediated accumulation of cyanidin derivatives in both strawberry leaves and fruits. Furthermore, FaTRAB1 forms a complex with FaMYB10 and FaTTG1 to participate in anthocyanin accumulation in strawberries. These findings provide valuable insights into the molecular basis of color variation in strawberry leaves.

## 1. Introduction

Fruit color, a key determinant of consumer preference, is primarily influenced by the types and concentrations of anthocyanins accumulated in the fruit [1]. As major products of the flavonoid pathway, anthocyanins mainly exist in six core structures in plants: cyanidin, pelargonidin, peonidin, petunidin, malvidin, and delphinidin [2]. Serving a dual biological function, these pigments act both as chromatic cues to attract pollinators and seed-dispersing animals [3] and as photoprotective compounds that mitigate light-mediated cellular damage in plants [4]. The health benefits of anthocyanins extend far beyond their traditional antioxidant and anti-inflammatory effects, with emerging evidence underscoring their critical function in improving glycemic control and insulin resistance [5]. Therefore, the molecular mechanisms of anthocyanin accumulation have been a major topic of research in the field of plant coloration.

The biosynthesis of anthocyanins in horticultural crops is well characterized, initiating from two key precursors: malonyl-CoA and coumaroyl-CoA [6]. The anthocyanin biosynthesis proceeds through sequential enzymatic reactions catalyzed by chalcone synthase (CHS), chalcone isomerase (CHI), flavanone 3-hydroxylase (F3H), flavonoid 3’-hydroxylase (F3’H), dihydroflavonol 4-reductase (DFR), and anthocyanidin synthase (ANS), yielding unstable anthocyanidin intermediates (pelargonidin, cyanidin, and delphinidin) [6,7]. These intermediates are then stabilized through glycosylation by glucosyltransferase, forming various anthocyanin derivatives including pelargonidin-3-*O*-glucoside (Pg3G), cyanidin-3-*O*-glucoside (C3G), and cyanidin-3,5-*O*-diglucoside (C3,5dG). Furthermore, O-methyltransferase (OMT) can modify C3G and C3,5dG to form peonidin derivatives, which are then glycosylated by glucosyltransferase to yield peonidin-3,5-*O*-diglucoside (Pg3,5dG) [8,9]. Multiple studies have demonstrated the conserved yet species-specific functions of MBW complexes (MYB, bHLH, and WD40-repeat proteins) in anthocyanin regulation. In *Malus domestica*, MdMYB308L-MdbHLH33 activates MdDFR, MdTTG1-MdbHLH3 [10,11], MdbHLH3 upregulates MdDFR/MdUFGT enhances pigmentation [12]. In pears and grape, PpbHLH64-PpMYB10 induces PpUFGT [13], VvMYBA2r-VvMYCA1-VvWDR1 activates anthocyanin genes [14].

Anthocyanin biosynthesis in strawberry is governed by intricate transcriptional networks and environmental cues. The FvHY5-FvbHLH9 heterodimer directly activates *FvDFR* to enhance pigmentation [15], while tissue-specific regulation occurs through distinct mechanisms: in ‘Ruegen’ petioles, the FvMYB10L-FvTT8 complex induces *FvDFR2* expression, yielding cyanidin and peonidin-derived purplish-red coloration, whereas fruits utilize both *FvDFR1* and *FvDFR2* to produce cyanidin and pelargonidin [16]. FvMYB10/FvMYB10L additionally regulate phenylpropanoid flux via *FvPAL1/2* activation, influencing fruit and petiole color phenotypes [17] Notably, FabHLH110 forms non-canonical MBW complexes with FaMYB10/90/114 to promote anthocyanins in fruit and petals of pink-flowered varieties independently of structural gene regulation [18], FaHY5 forms a complex with FaCRY1-FaCOP1 to promote anthocyanin accumulation in callus tissues [19], while FaMYB5 positively modulates fruit pigmentation [20] and FaMYB1 suppresses cyanidin 3-rutinoside and quercetin-glycosides in transgenic tobacco [21]. Environmental modulation occurs through cold-induced FvMAPK3 activation, which phosphorylates FvMYB10 and destabilizes *FvCHS1* to inhibit anthocyanin accumulation [22].

In addition to MBW-type transcription factors, ERF, B-box, RAV, and bZIP transcription factors also serve as pivotal regulators of plant anthocyanin biosynthesis. For example, MdERF109 directly targets to the promoters of *MdbHLH3*, *MdCHS*, and *MdUFGT* [23]; MaERF5 interacts with MaMYBA and MaF3H [24]; Pp12ERF96 and Pp4ERF24 interact with PpMYB114 [25], VvFHY3-VvbZIP17 regulated expression of anthocyanin structural genes [26], collectively promoting anthocyanin accumulation in apple, mulberry, grape and pear fruits. In *Malus spectabilis*, MsERF17 specifically activates the expression of *MsbHLH3* and *MsF3’H*, enhancing leaf anthocyanin production [27]. Similarly, the strawberry B-box TF FaBBX22 upregulates multiple structural genes (FaPAL, FaANS, FaF3’H, FaUFGT1) and the transporter gene FaRAP to promote anthocyanin accumulation in callus tissues [28]. The RAV (related to ABI3/viviparous 1) TF FaRAV1, which stimulates anthocyanin biosynthesis both through direct activation of pathway genes and by inducing FaMYB10 expression [29]. Complementary mechanisms exist in tomato, where SlAREB1 directly targets *SlDFR* and *SlF3’5’H* promoters to modulate seedling pigmentation [30].

The basic region/leucine zipper (bZIP) transcription factors in plants modulate various physiological processes, ranging from pathogen resistance and stress/light responses to seed maturation and flower development. In Arabidopsis, the 78 AtbZIP transcription factors are classified into 13 subfamilies (A-I, S, M, K, and J) [31]. Currently, 54 bZIP genes have been identified in woodland strawberry (*Fragaria vesca*), which are divided into 10 subfamilies (A-I and S) [32]. In strawberry, the A subfamily member FvABF3, functioning as an ABA signaling response factor, forms a cascade with FvALKBH10B and FvSEP3 to regulate fruit ripening [33]. bZIP transcription factors play conserved yet functionally diverse roles in regulating anthocyanin biosynthesis across plant species. For example, in sweet cherry, lotus, and ‘pinkspire’, PavbZIP6, NnbZIP36, and MpbZIP9 enhance pigmentation by regulating anthocyanin structural genes (*F3H*, *F3’H*, *DFR*, *ANS*, and *UFGT*) [34–36]. In apple, MdbZIP44 and MdbZIP23 interact with MdMYB1 and MdNAC1, respectively, indirectly regulating *MdDFR* and *MdUFGT* promotes anthocyanin production [37,38]. Additionally, overexpression of *MdHY5* or knockout of *VvbZIP3* activates anthocyanin structural genes to enhance pigmentation [39,40]. Meanwhile, PybZIPa primarily regulates transcriptional activators (PyMYB114, PyMYB10, PyBBX22) and PyUFGT to promote pigment accumulation [41]. Previous studies have demonstrated that in strawberry, FaHY5 upregulates the promoter activity of FaRAP, while FvHY5 directly activates *FvDFR* expression to enhance pigmentation [15,19]. However, the broader functional involvement of bZIP transcription factors in regulating strawberry anthocyanin biosynthesis remains largely unexplored.

The fruit of cultivated strawberries predominantly presents a red color due to the accumulation of Pg3G [42]. The wild-type ‘Ruegen’ strawberry fruit, however, mainly contains cyanidin and appears slightly purplish [43]. Its petioles also appear purplish-red due to the presence of cyanidin and peonidin [16]. Recent investigations have established that mutating the arginine in F3H to histidine leads to pink-colored ‘Ruegen’ strawberry fruits [44], but it has not been reported whether anthocyanins accumulate in strawberry leaves. There, we discovered a novel mechanism of anthocyanin accumulation in ‘Ruegen’ strawberries leaves, which is different from the mechanism in strawberry fruit. The bZIP transcription factor FaTRAB1 may compete with FabHLH3 to bind FaMYB10 and FaTTG1, thereby forming a new complex that synergistically activates *FaF3’H*, *FaANS*, *FaUFGT*, and *FaOMT*, primarily promoting the accumulation of cyanidin and peonidin in the leaves. This study revealed the molecular regulatory mechanism of anthocyanin accumulation in strawberry leaves, providing new insights for the creation of new strawberry germplasm with different types and contents of anthocyanins.

## 2. Result

### 2.1 Identification of FaTRAB1

**Fig 1:**
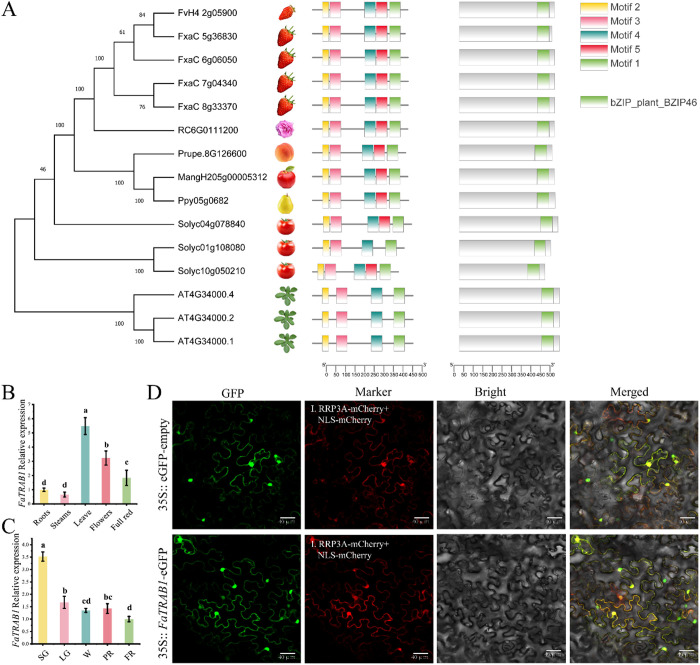
Identification of FaTRAB1. Phylogenetic relationships **(A)**. *FaTRAB1* tissue-specific (B) and developmental stage expression profile **(C)**. SG, small green; LG, large green; W, white fruit stage; PR, partial red; FR, full red stage. Subcellular localization of FaTRAB1(D). (I) cytosolic marker RPP3A-mCherry and nucleus marker NLS-mCherry. The scale bar represents 40 µm. Different letter symbol indicates a statistically significant difference at *p* ≤ 0.05.

Phylogenetic tree, domain, and motif analyses of transcription responsible for ABI and ABRE binding (FxaC7g04340) and its closely related TRAB proteins from species such as wild strawberry (FvH4 2g05900, FvTRAB1) and rose (RC6G0111200) indicate that FxaC7g04340 is closely related to these TRAB proteins, therefore, FxaC7g04340 was designated as FaTRAB1. Based on conserved sequence alignment, the cultivated strawberry FaTRAB1 is homologous to *Arabidopsis* AtABF3 (AT4G34000.1) and belongs to the A subfamily of the bZIP gene family. At the same time, these TRAB proteins all contain five identical motifs and a typical bZIP-plant-BZIP46 domain (Fig 1A). FaTRAB1 is highly expressed in strawberry leaves and flowers, followed by fully red (FR) fruits, roots, and stems (Fig 1B). The expression level of FaTRAB1 gradually decreases as the fruit matures, with the highest expression observed during the small green (SG) fruit stage (Fig 1C). Subcellular localization results showed that FaTRAB1 is expressed in both the cytoplasm and the nucleus (Fig 1D).

### 2.2. Stable overexpression of *FATRAB1* promotes anthocyanin and chlorophyll accumulation in ‘Ruegen’ strawberry leaves

**Fig 2:**
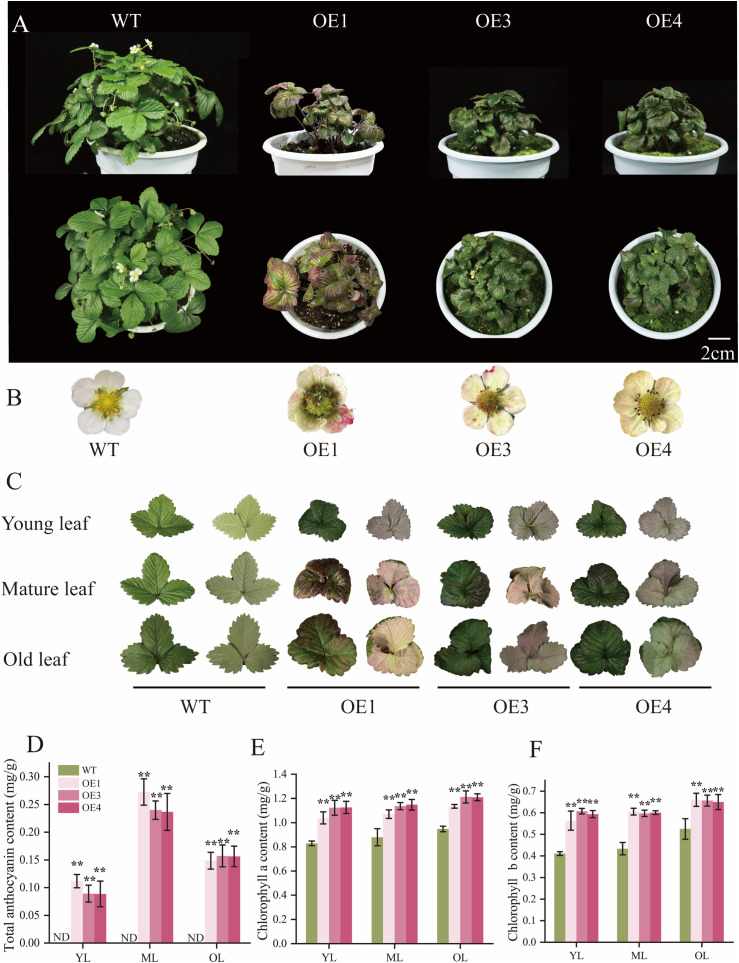
*FaTRAB1* promotes anthocyanin and chlorophyll accumulation in strawberry leaves. Phenotypes of WT and *FaTRAB1* overexpression strawberry plants **(A)**, phenotypes of flowers (B) and leaves accumulating anthocyanins in WT and *FaTRAB1* overexpression strawberries **(C)**, total anthocyanin content in leaves **(D)**, and chlorophyll a and b content in leaves **(E and F)**. WT: wild type, OE: overexpressed *FaTRAB1*. The scale bar in Figs 1A is 2 cm. Statistical significance is indicated as follows: *p ≤ 0.05, **p ≤ 0.01. The same below.

To study *FaTRAB1*’s role in strawberry development, we generated stable *FaTRAB1*-overexpressed transgenic ‘Ruegen’ lines. Based on GUS staining and RT-qPCR results, we selected overexpression lines 1, 3, and 4 with deeper staining and higher expression levels for further cultivation and observation (S1A Fig and B Fig). Compared to WT, the leaves of OE1, OE3, and OE4 lines appeared greener, with varying degrees anthocyanin accumulation on upper and lower surfaces of leaves, and petals. (Fig 2A-C). Further measurements of total anthocyanin and chlorophyll content in young leaves, mature leaves, and senescent leaves from WT and overexpression lines revealed that overexpressing FaTRAB1 stimulates coordinated accumulation of anthocyanins and chlorophyll in strawberry leaves. The anthocyanin content showed an increasing-decreasing trend during leaf development, reaching up to 0.27 mg/g in mature leaves (Fig 2D), while no anthocyanin accumulation was observed in wild-type plant leaves. FaTRAB1 overexpression significantly increased chlorophyll a (22%) and b (33%) content in strawberry leaves compared to WT (Fig 2D-F). Results identify *FaTRAB1* as a bifunctional regulator of leaf chlorophyll and anthocyanin production.

### 2.3. *FaTRAB1* promotes chloroplast development in strawberry leaves

**Fig 3:**
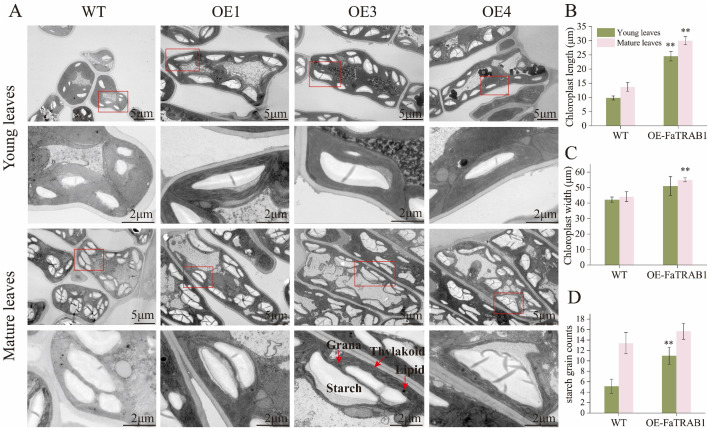
Observation of chloroplast structure in strawberry leaves (A). Chloroplast Length (B), Chloroplast Width (C), Number of Starch Grains (D). Starch grains are indicated within the red box. WT: wild type, OE: overexpressed FaTRAB1.Statistical significance is indicated as follows: *p ≤ 0.05, **p ≤ 0.01.

To preliminarily explore why overexpressing *FaTRAB1* promotes chlorophyll accumulation in strawberry leaves, we used transmission electron microscopy to observe structural changes in the chloroplasts of strawberry leaves. Compared to WT, the granum and thylakoid arrangements in the leaves of the OE-*FaTRAB1* lines were more compact (Fig 3A), and the chloroplasts in their mature leaves were significantly larger, with lengths and widths measuring 16.25 μm and 10.8 μm, respectively (Fig 3B and C). Additionally, within individual chloroplasts, the number of starch grains in young leaves of the OE-*FaTRAB1* lines was twice that of the WT, and there was also an increasing trend in the number of starch grains in mature leaves (Fig 3D). These results suggest that overexpressing *FaTRAB1* promotes chloroplast development.

### 2.4. Analysis of anthocyanin types and content in strawberry leaves overexpressing *FaTRAB1*

**Fig 4:**
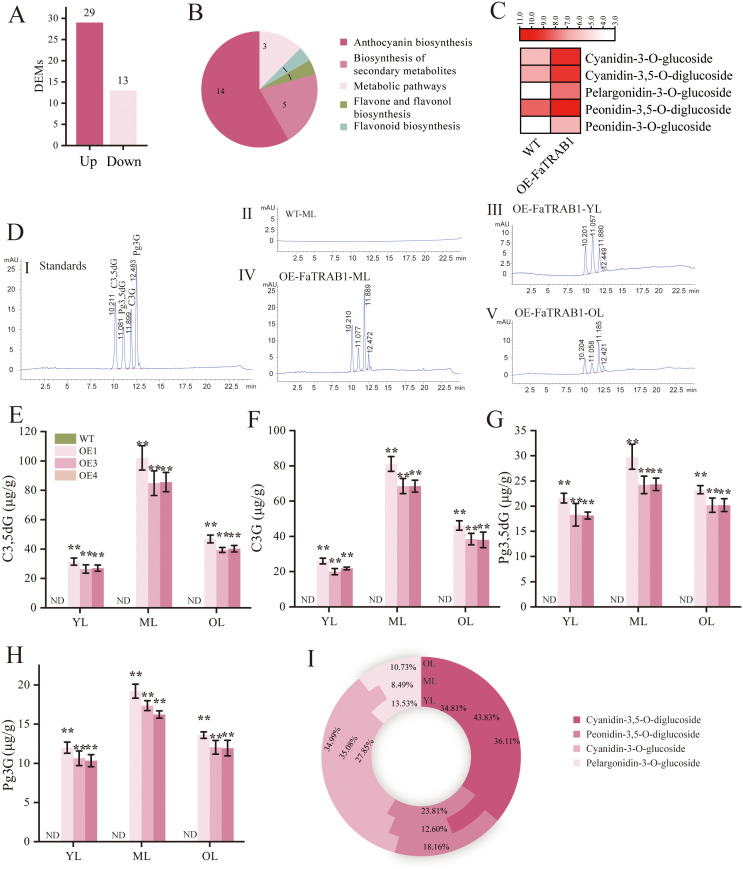
Mass spectrometry analysis and HPLC determination of anthocyanins in strawberry leaves. Differential metabolite statistics **(A)**, KEGG enrichment analysis of differential metabolites **(B)**, heatmap of relative content of five anthocyanin mass spectrometry analysis **(C)**, with anthocyanin content presented in log_2_ scale, the values of the metabolites with log_2_FC < 0.5. HPLC analysis of different anthocyanins **(D)** (I: peak times for the four anthocyanin standards: Cyanidin-3,5-*O*-diglucoside (C3,5dG, peak time approximately 10.21 min), Peonidin-3,5-*O*-diglucoside (Pg3,5dG, peak time approximately 11.08 min), Cyanidin-3-*O*-glucoside (C3G, peak time approximately 11.89 min), Pelargonidin-3-*O*-glucoside (Pg3G, peak time approximately 12.48 min); II: HPLC peak chart for WT leaves; III: HPLC peak chart for OE-*FaTRAB1* young leaves; IV: HPLC peak chart for OE-*FaTRAB1* mature leaves; V: HPLC peak chart for OE-*FaTRAB1* old leaves. Content of C3,5dG **(E)**. Content of C3G **(F)**. Content of Pg3,5dG **(G)**. Content of Pg3G **(H)**. Proportion of C3,5dG, Pg3,5dG, C3G, and Pg3G in young leaves, mature leaves, and old leaves of OE-*FaTRAB1* **(I)**. YL: young leaf; ML: mature leaf; OL: old leaf. WT: wild type, OE: overexpressed *FaTRAB1*. Statistical significance is indicated as follows: *p ≤ 0.05, **p ≤ 0.01.

To clarify composition and concentration changes of anthocyanins in strawberry leaves overexpressing *FaTRAB1*, we performed mass spectrometry analysis on the mature leaves with the highest anthocyanin accumulation. The mass spectrometry results identified a total of 42 differential expression metabolites (DEMs), 29 showed significant upregulation while 13 were downregulated relative to WT (Fig 4A). These DEMs including 17 types of cyanidin, 9 types of pelargonidin, 8 types of peonidin, 5 types of delphinidin, 1 type of quercetin, 1 type of malvidin, and 1 type of petunidin (Table A in S1 Text). KEGG enrichment analysis revealed that 14 metabolites were enriched in anthocyanin biosynthesis and 5 in secondary metabolite biosynthesis pathways (Fig 4B). Further, we quantified anthocyanin composition and concentration (threshold: > 50 μg/g in OE lines, > 10 μg/g in WT) (Fig 4C). Subsequently, we quantified anthocyanin composition in strawberry leaves at three developmental stages (young/mature/old) using HPLC. Four anthocyanins—cyanidin-3,5-*O*-diglucoside (C3,5dG), cyanidin-3-*O*-glucoside (C3G), peonidin-3,5-*O*-diglucoside (Pg3,5dG), and pelargonidin-3-*O*-glucoside (Pg3G)—were detected in the leaves of OE1, OE3, and OE4 plants. Their content showed a trend of increasing and then decreasing as the leaves developed, but no anthocyanins were detected in the WT (Fig 4D). In young leaves, mature leaves, and old leaves, the contents of C3,5dG in OE1, OE3, and OE4 were 31.453 μg/g, 102.048 μg/g, and 46.890 μg/g, respectively; C3G content was 26.018 μg/g, 81.124 μg/g, and 46.175 μg/g, respectively; Pg3,5dG content was 21.594 μg/g, 29.802 μg/g, and 23.262 μg/g, respectively; and Pg3G content was 12.003 μg/g, 19.205 μg/g, and 13.597 μg/g, respectively. The highest content of these four metabolites was found in mature leaves (Fig 4E–H). Similarly, we analyzed the proportions of these four anthocyanins in young leaves, mature leaves, and old leaves. The results showed that cyanidin types (C3,5dG and C3G) had the highest proportions of 62.66%, 78.91%, and 71.1%, respectively, followed by Pg3,5dG with proportions of 23.81%, 12.6%, and 18.16%, and the lowest proportion was Pg3G with 10.73%, 8.49%, and 13.53%, respectively (Fig 4I). These results suggest that overexpressing *FaTRAB1* leads to the accumulation of more cyanidin and peonidin anthocyanins in strawberry leaves, accompanied by a small amount of pelargonidin accumulation, ultimately resulting in leaves exhibiting a reddish-purple color.

### 2.5. Transcriptome analysis of mature strawberry leaves overexpressing *FaTRAB1*

**Fig 5:**
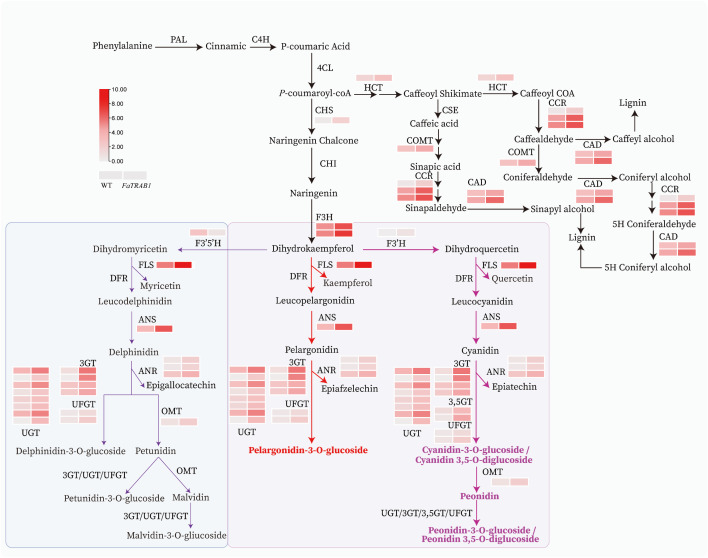
Phenylpropanoid biosynthesis pathway. Heatmap visualization used log_2_(FPKM) values, FPKM value with log2FC < 0.5. Phenylalanine ammonia-lyase (PAL), cinnamate 4-hydroxylase (C4H), 4-Coumarate-CoA ligase (4CL), chalcone synthase (CHS), chalcone isomerase (CHI), flavanone 3-hydroxylase (F3H), flavonol synthase (FLS), flavonoid 3’-hydroxylase (F3’H), flavonoid 3’,5’-hydroxylase (F3’5’H), dihydroflavonol 4-reductase (DFR), anthocyanidin synthase (ANS), anthocyanidin reductase (ANR), UDP-glucose: flavonoid 3-*O*-glucosyltransferase (UFGT), anthocyanidin 3-*O*-glucosyltransferase (3GT), anthocyanidin 3,5-*O*-diglucosyltransferase (3,5GT), UDP-glycosyltransferase (UGT), O-Methyltransferase (OMT), hydroxycinnamoyl-CoA shikimate/quinate transferase (HCT), cinnamoyl-CoA reductase (CCR), caffeoyl shikimate esterase (CSE), cinnamyl alcohol dehydrogenase (CAD).

To elucidate *FaTRAB1*-mediated anthocyanin accumulation, we conducted RNA-seq analysis of mature leaves from OE1 and WT. Compared to WT, 5296 differentially expressed genes (DEGs) in OE-*FaTRAB1* leaves (2923 upregulated, 2373 downregulated) (S2A Fig). KEGG enrichment analysis of all DEGs revealed that 66.1% of the DEGs were enriched in metabolism pathways, mainly including starch and sucrose metabolism and phenylpropanoid biosynthesis (S2B Fig). Further analysis of the phenylpropanoid biosynthesis pathway revealed that the transcript levels of structural genes in this pathway were significantly upregulated in OE-*FaTRAB1*, including CHS, F3H, F3’H, FLS, ANS, 3GT/3,5GT/UFGT/UGT, ANR, and OMT (Table B in S1 Text). However, F3’5’H transcript levels were significantly downregulated. This suggests that *FaTRAB1* may activate the transcription of F3’H but suppress the transcription of F3’5’H, leading to more dihydrokaempferol being converted into cyanidin-type anthocyanin biosynthesis pathways in strawberry leaves (Fig 5), resulting in the production of more cyanidin in the leaves of OE-*FaTRAB1* plants, which is subsequently converted into stable C3G and C3,5dG by glycosyltransferase to give color. Importantly, FaTRAB1 may also activate FaOMT to promote the conversion of C3G and C3,5dG into Peonidin, which is further glycosylated into Pg3,5dG (Fig 5), thus accumulating peonidin in the strawberry leaves. Furthermore, beyond anthocyanin accumulation, *FaTRAB1* overexpression increased lignin content, concomitant with elevated transcript levels of lignin biosynthetic genes (hydroxycinnamoyl-CoA shikimate/quinate transferase (HCT), cinnamoyl-CoA reductase (CCR), cinnamyl alcohol dehydrogenase (CAD), and COMT) (Fig 5). Subsequently, we conducted a quantitative analysis of the structural genes in the phenylpropanoid biosynthesis pathway (*PAL*, *C4H*, *4CL*, *F3H*, *F3’H*, *DFR*, *ANS*, *UFGT*, *OMT*) and strawberry anthocyanin synthesis-related transcription factors (bHLH3, TTG1, MYB10).

RT-qPCR analysis revealed significant upregulation of target genes in OE-*FaTRAB1 l*eaves across all developmental stages (young, mature, old) versus WT, with peak expression observed in mature leaves (S3 Fig).

### 2.6. FaTRAB1 positively regulates the promoters of *FaF3’H*, *FaANS*, *FaUFGT*, and *FaOMT*

**Fig 6:**
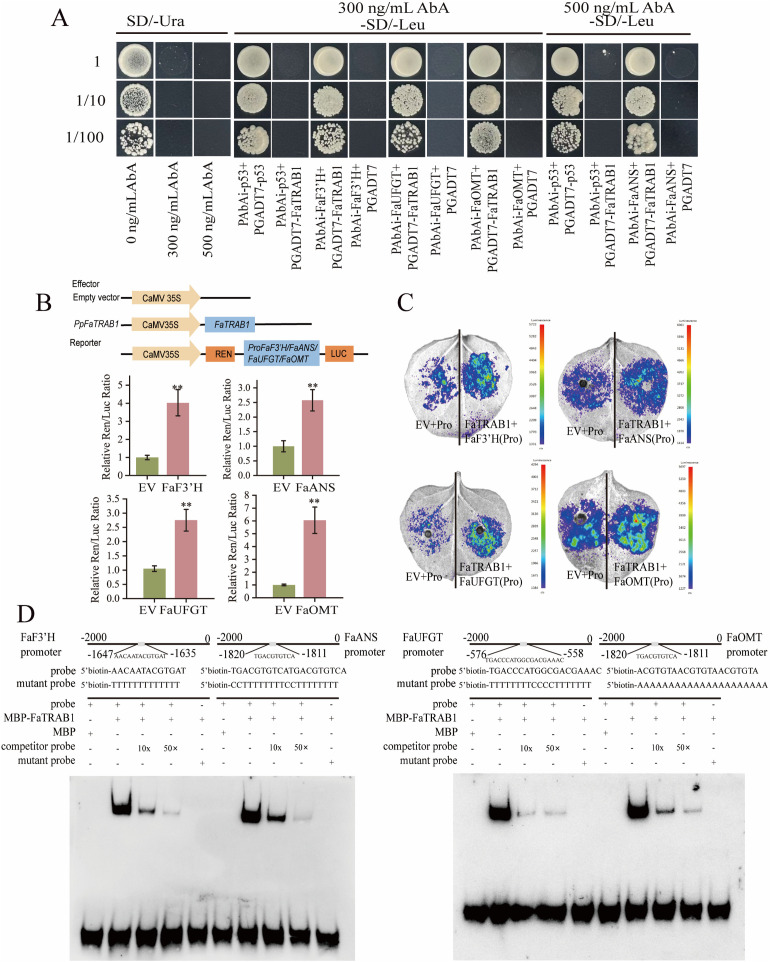
FaTRAB1 can bind to the promoter of *FaF3’H, FaANS, FaUFGT,* and *FaOMT.* Yeast one-hybrid (Y1H) **(A)**, dual-luciferase assay **(B)**, fluorescence images **(C)**, electrophoretic mobility shift assay (EMSA) **(D)**. Ab: pAbAi; AD: pGADT7. Positive control: pAbAi-p53 + pGADT7-p53, negative control: pAbAi-p53 + pGADT7-*FaF3’H/*pGADT7-*FaANS*/pGADT7-*FaUFGT*/pGADT7-*FaOMT*, pAbAi-*FaF3’H*/pAbAi-*FaANS*/pAbAi-*FaUFGT*/pAbAi-*FaOMT* + pGADT7. EV: 35S empty and pGreen-*FaF3’H/FaANS/FaUFGT/FaOMT* co-injected tobacco, *FaF3’H/FaANS/FaUFGT/FaOMT*: 35S::FaTRAB1 and pGreen-*FaF3’H/FaANS/FaUFGT/FaOMT* co-injected tobacco. Statistical significance is indicated as follows: **p ≤ 0.01.

Transcriptomic and RT-qPCR analyses consistently showed that *FaTRAB1* overexpression specifically upregulates late anthocyanin biosynthetic genes (*ANS, UFGT, OMT*) in mature leaves (S3 Fig). Additionally, the accumulation of cyanidin in strawberry leaves was substantial, and *ANS, UFGT, OMT*, and *F3’H* were identified as candidate target genes regulated by *FaTRAB1*. Our analysis revealed that FaTRAB1 could potentially regulate the promoters of *FaF3’H* (cis-element: AACAATACGTGAT), *FaANS* (cis-element: TGACGTGTCA), *FaUFGT* (cis-element: TGACCCATGGCGACGAAAC), and *FaOMT* (cis-element: ACGTGTA). Yeast one-hybrid (Y1H) assays demonstrated that while the positive control (pAbAi-p53 + pGADT7-p53), negative controls (pAbAi-p53 + pGADT7-FaTRAB1, pAbAi-FaF3’H/pAbAi-FaANS/pAbAi-FaUFGT/pAbAi-FaOMT + pGADT7), and experimental groups (pAbAi-FaF3’H/pAbAi-FaANS/pAbAi-FaUFGT/pAbAi-FaOMT + pGADT7-FaTRAB1) all grew normally on SD/-Leu medium (Fig 6A), the positive control and experimental groups grew on SD/-Leu/300 (500) ng/mL AbA medium. This selective growth confirmed FaTRAB1’s specific binding to the promoter regions of *FaF3’H, FaANS, FaUFGT*, and *FaOMT*.

Dual-luciferase reporter assays and in vivo imaging further verified *FaTRAB1*’s positive regulatory effect on these promoters (Fig 6B). In the dual-luciferase in vivo imaging experiments, the experimental groups (FaTRAB1 + pGreen-FaGAPC2/pGreen-FaANS/pGreen-FaUFGT/pGreen-FaOMT) exhibited significantly stronger fluorescence at the injection sites compared to controls (Fig 6C), providing conclusive evidence that FaTRAB1 transcriptionally activates *FaF3’H, FaANS, FaUFGT,* and *FaOMT*. Finally, EMSA was performed using a biotin-labeled probe. When incubated with MBP-FaTRAB1 fusion protein, we observed that the probe migrated from the *FaF3’H, FaANS, FaUFGT*, and *FaOMT* promoter regions. Migration was reduced after the addition of competing probes, while no migration was detected by probes containing mutant sequences in these regions (Fig 6D). These experiments showed that FaTRAB1 binds to sequences in the *FaF3’H, FaANS, FaUFGT*, and *FaOMT* promoters.

### 2.7. FaTRAB1 interacts with FaMYB10 and FaTTG1

**Fig 7:**
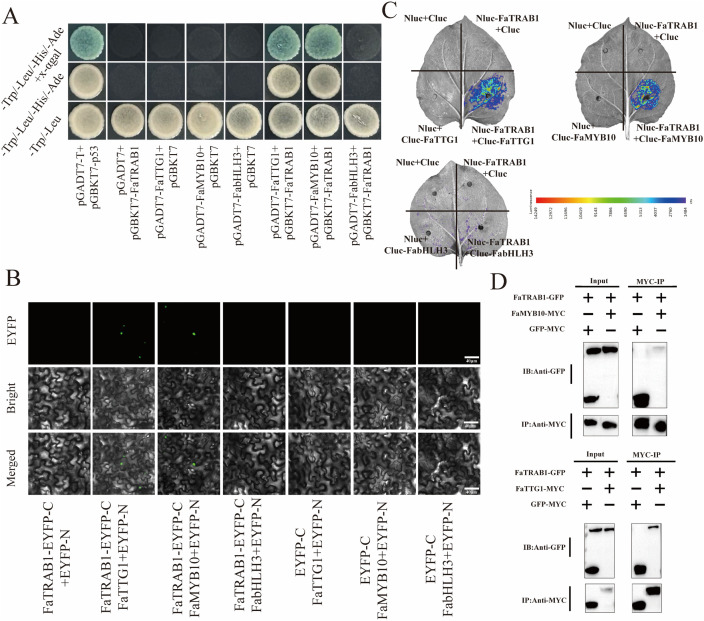
FaTRAB1 interact with FaTTG1 and FaMYB10. Y2H analysis(A). Positive control: pGADT7-T + pGBKT7-p53; negative control: pGADT7-empty+ pGBKT7-FaTRAB1, pGBKT7-empty + pGADT7-FaTTG1/FaMYB10/FabHLH3. BiFC analysis **(B)**, negative control: FaTRAB1-EYFP-C+EYFP-N, EYFP-C + FaTTG1/FaMYB10/FabHLH3-EYFP-N. The firefly luciferase complementation imaging (LCI) assay **(C)**, negative control: nLUC-FaTRAB1 + cLUC, cLUC-FaTTG1/ FaMYB10/FabHLH3 + nLUC **(C)**. Co-Immunoprecipitation Analysis (Co-IP) **(D)**.

To verify whether FaTRAB1 interacts with the MBW complex (FaMYB10, FaTTG1, and bHLH3). We conducted a pairwise yeast two-hybrid assay with FaTRAB1, FaTTG1, FaMYB10, and FabHLH3. The control group (pGADT7-T + pGBKT7-p53, pGADT7-empty + pGBKT7-FaTRAB1, pGBKT7-empty + pGADT7-FaTTG1/FaMYB10/FabHLH3) and experimental group (pGBKT7-FaTRAB1 + pGADT7-FaTTG1/pGADT7-MYB10/pGADT7-bHLH3) both grew on SD-Trp/-Leu medium. However, on SD/-Trp/-Leu/-His/-Ade and SD/-Trp/-Leu/-His/-Ade + x-α-gal media, the positive control (pGADT7-T + pGBKT7-p53) and experimental groups (pGBKT7-FaTRAB1 + pGADT7-FaTTG1 and BD-FaTRAB1 + pGADT7-MYB10) grew normally and activated the substrate x-α-gal for α-galactosidase (MEL1) to turn the colonies blue, whereas the experimental group pGBKT7-FaTRAB1 + pGADT7-bHLH3 did not turn blue (Fig 7A). This result suggests that FaTRAB1 interacts with FaTTG1 and MYB10, but not with bHLH3.

To further confirm the interactions between FaTRAB1 and FaTTG1, FaMYB10, and FabHLH3, we performed BIFC and LCI assays. In the BIFC assay, the co-expression control group: FaTRAB1-EYFP-C + EYFP-N, EYFP-C + FaTTG1/FaMYB10/FabHLH3-EYFP-N did not show green fluorescence signals in tobacco cells, while the co-expression experimental groups FaTRAB1-EYFP-C + FaTTG1 and FaTRAB1-EYFP-C + FaMYB10 exhibited green fluorescence in the cytoplasm of tobacco epidermal cells. The experimental group FaTRAB1-EYFP-C + bHLH3-EYFP-N did not show green fluorescence in the tobacco epidermal cells (Fig 7B), confirming that FaTRAB1 interacts with FaTTG1 and FaMYB10, but not with FabHLH3.

In the LCI assay, the negative control group (nLUC-FaTRAB1 + cLUC and cLUC-FaTTG1/cLUC-FaMYB10/cLUC-FabHLH3 + nLUC) did not show fluorescence in tobacco, while the co-injection experimental groups (nLUC-FaTRAB1 + cLUC-FaTTG1, nLUC-FaTRAB1 + cLUC-FaMYB10) showed fluorescence at the injection site. The experimental group cLUC-FaTRAB1 + cLUC-FabHLH3 did not show fluorescence at the injection site (Fig 7C), further confirming that FaTRAB1 interacts with FaTTG1 and FaMYB10, but not with FabHLH3. These interaction results are consistent with the Y2H results, further validating the interaction between FaTRAB1 and FaTTG1 and FaMYB10, but not with bHLH3. Finally, by co-expressing GFP-FaTRAB1 and MYC-FaMYB10, as well as GFP-FaTRAB1 and MYC-FaTTG1, in tobacco leaves, co-immunoprecipitation (Co-IP) assays were performed to confirm the interaction between FaTRAB1 and FaMYB10/FaTTG1 (Fig 7D).

### 2.8. The effect of *FaTRAB1* on anthocyanin key structural genes in strawberry fruit coloration

**Fig 8:**
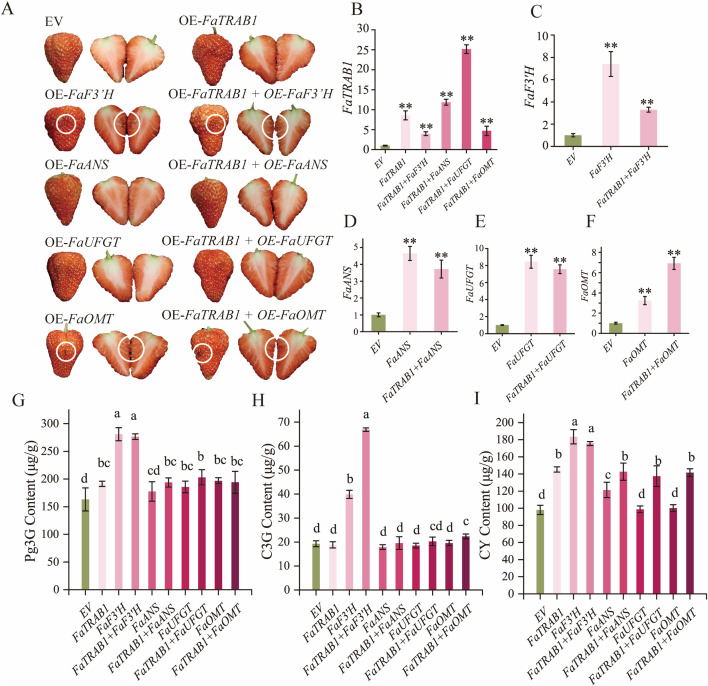
Transient overexpression of *FaTRAB1, FaF3’H, FaANS, FaUFGT*, and *FaOMT* in strawberry fruit. Strawberry fruit phenotypes **(A)**. EV: 35S empty vector injection, OE: overexpression group. Relative expression levels of *FaTRAB1* **(B)**, *FaF3’H* **(C)**, *FaANS* **(D)**, *FaUFGT* **(E)**, *FaOMT* **(F)**, pelargonidin-3-*O*-glucoside (Pg3G) **(G)**, cyanidin-3-*O*-glucoside (C3G) **(H)**, cyanidin (CY) **(I)**. Statistical significance is indicated as follows: **p ≤ 0.01. Different letter symbols indicate a statistically significant difference at *p* ≤ 0.05.

To further explore the effect of *FaTRAB1* on anthocyanin key structural genes in strawberry fruit coloration, we conducted transient overexpression and co-expression of *FaTRAB1, FaF3’H, FaANS, FaUFGT, FaOMT*, and other anthocyanin key structural genes in ‘Red Yan’ strawberry fruits. The results showed that transient overexpression of *FaTRAB1, FaANS, FaUFGT* alone or co-expression of *FaTRAB1* + *FaANS* and **FaTRAB1* *+ *FaUFGT* did not lead to visible anthocyanin accumulation in strawberry (*Fragaria* × *ananassa* Duch.) fruits. However, fruits that overexpressed FaF3’H or *FaOMT* alone, or co-expressed *FaTRAB1* + *FaF3’H* or *FaTRAB1* + *FaOMT*, showed significant anthocyanin accumulation (Fig 8A). RT-qPCR results confirmed successful overexpression of *FaTRAB1, FaF3’H, FaANS, FaUFGT,* and *FaOMT* in strawberry fruits (Fig 8B–F). HPLC analysis indicated that transient overexpression of *FaF3’H, FaTRAB1* + *FaF3’H*, and *FaTRAB1* + *FaOMT* significantly increased the contents of Pg3G, C3G, and CY in strawberry fruits compared to the control, while overexpression of *FaOMT* only increased the contents of Pg3G and C3G (Fig 8G–I). In the treatments with transient overexpression of *FaTRAB1, FaANS, FaUFGT*, or co-expression of *FaTRAB1* + *FaANS/FaUFGT*, we observed a significant increase in Pg3G content compared to the control, but no significant difference in C3G content (Fig 8G and H). Additionally, the CY content was also significantly increased in the transient overexpression of *FaTRAB1, FaANS*, or co-expression of *FaTRAB1* + *FaANS/FaUFGT* treatment groups (Fig 8I). Furthermore, co-expression of *FaTRAB1* +* *FaANS/FaUFGT/FaOMT** significantly increased the CY content in fruits compared to transient overexpression of *FaANS/FaUFGT/FaOMT* alone (Fig 8G–I). It is noteworthy that the Pg3G, C3G, and CY contents in the *FaTRAB1* + *FaF3’H* co-expression group were 1.45, 3.55, and 1.21-folds higher, respectively, than those in the group with *FaTRAB1* alone. Compared to the transient overexpression of FaF3’H alone, the C3G content in the **FaTRAB1* *+ F*aF3’H* co-expression group was significantly increased 1.68-fold. These results indicate that FaTRAB1, as an upstream regulatory factor, promotes the accumulation of the unstable anthocyanin CY by enhancing the expression of *FaF3’H*, *FaANS*, and *FaUFGT*; meanwhile, *FaF3’H* directs metabolic flux toward the synthesis of C3G. Co-expression of **FaTRAB1* *+ *FaF3’H* significantly amplifies the catalytic effect of *FaF3’H*, thereby establishing an efficient and stable C3G biosynthesis pathway. Therefore, *FaF3’H* may be the key gene determining FaTRAB1-mediated regulation of cyanidin-derived anthocyanin accumulation.

### 2.9. *FaTRAB1* forms a new complex with *FaMYB10* and *FaTTG1* to regulate anthocyanin accumulation

**Fig 9:**
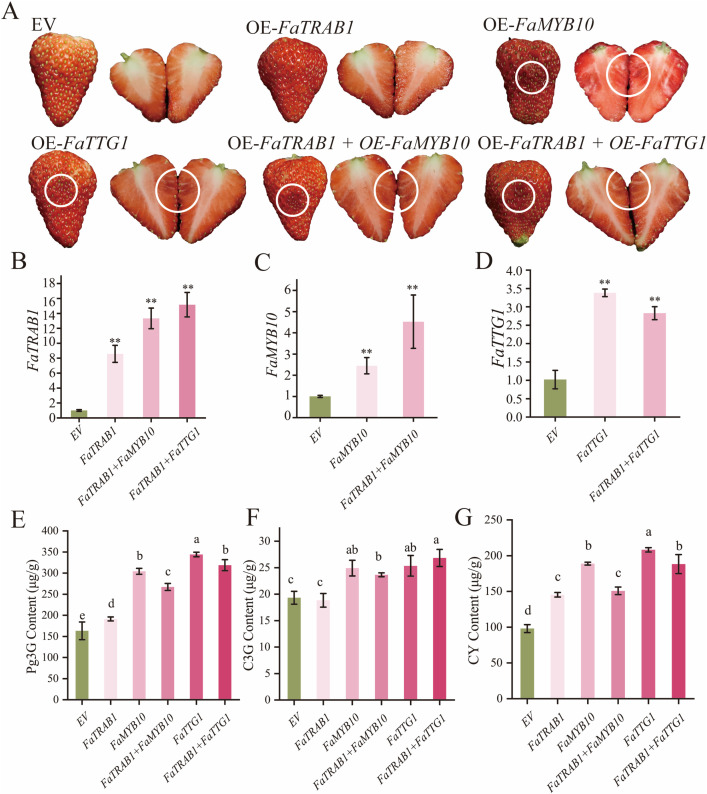
Transient overexpression of *FaTRAB1, FaMYB10,* and *FaTTG1* in strawberry. Strawberry fruit phenotypes **(A)**. EV: 35S empty vector injection, OE: overexpression group. Relative expression levels of *FaTRAB1* **(B)**, *FaMYB10* **(C)**, *FaTTG1* **(D)**, pelargonidin-3-*O*-glucoside (Pg3G) **(E)**, cyanidin-3-*O*-glucoside (C3G) **(F)**, cyanidin (CY) **(G)**. Statistical significance is indicated as follows: **p ≤ 0.01. Different letter symbols indicate a statistically significant difference at *p* ≤ 0.05.

Based on the results of the interaction between *FaTRAB1*, *FaMYB10*, and *FaTTG1*, we further conducted transient overexpression of *FaTRAB1, FaMYB10, FaTTG1*, and co-overexpression of **FaTRAB1* *+* *FaMYB10/FaTTG1** in strawberries to investigate the role of *FaTRAB1* in synergistically regulating anthocyanin accumulation with *FaMYB10* and *FaTTG1*. We then observed the anthocyanin accumulation at the injection sites and found that, except for the transient overexpression of *FaTRAB1* alone, overexpression of *FaMYB10, FaTTG1*, and co-overexpression of **FaTRAB1* *+ *FaMYB10/FaTTG1* all promoted anthocyanin accumulation in the fruit (Fig 9A). RT-qPCR analysis confirmed successful overexpression of *FaTRAB1*, *FaMYB10*, and *FaTTG1* in strawberry fruits (Fig 9B–D). Compared to the control, overexpression of *FaMYB10, FaTTG1,* and co-overexpression of *FaTRAB1* + *FaMYB10/FaTTG1* significantly increased the contents of Pg3G, C3G, and CY in strawberry fruits (Fig 9E-G). Compared to the transient overexpression of *FaMYB10* or *FaTTG1* alone, co-overexpression of **FaTRAB1* *+ *FaMYB10/FaTTG1* significantly decreased the levels of Pg3G and CY in the fruits (Fig 9E and G). Therefore, the formation of a new complex between *FaTRAB1* and *FaMYB10/FaTTG1* may inhibit the effect of *FaMYB10* or *FaTTG1* in promoting anthocyanin accumulation.

## 3. Discussion

In recent years, the role of bZIP transcription factors in regulating anthocyanin synthesis in plants has been widely studied. Research in various species has shown that bZIP family members, such as NnbZIP36 in lotus [35], MpbZIP9 and MdbZIP44 in apple [36,37], VvbZIP36 in grape [40], FvHY5 and FaHY5 in strawberry [15,19] promote anthocyanin accumulation primarily by regulating the expression of *CHI, F3’H, DFR, ANS*, and *UFGT*. However, the precise regulatory roles and molecular mechanisms of bZIP transcription factors in strawberry anthocyanin biosynthesis require further investigation.

### 3.1. The new function of *FaTRAB1*: promotes anthocyanin accumulation in strawberry leaves

The bZIP transcription factor FaTRAB1 exhibits high expression in the leaves and small green fruits of strawberries (Fig 1B and C). By stably expressing *FaTRAB1* in ‘Ruegen’ strawberries, we observed that the gene significantly promoted the accumulation of reddish-purple anthocyanins in the leaves and petals. The MS results showed that OE-FaTRAB1 primarily promoted the accumulation of cyanidin derivatives (50.21%) and peonidin derivatives (40.85%). Among these, the content of peonidin-3,5- *O* -diglucoside (Pg3,5dG) was the highest (1026.8 μg/g), followed by cyanidin-3- *O* -glucoside (C3G, 751.21 μg/g) and cyanidin-3,5- *O* -diglucoside (C3,5dG, 620.78 μg/g). HPLC analysis showed that cyanidin derivatives (C3,5dG and C3G) were the most abundant in the overexpression lines (62.66%-78.91%), followed by Pg3,5dG (12.6%-23.81%), with Pg3G being the least abundant (8.49%-13.53%) (Fig 4I). However, in the HPLC analysis, the measured content of Pg3,5dG was not the highest. This discrepancy may arise because MS detected all existing forms of Pg3,5dG with high sensitivity, including: free Pg3,5dG, acylated derivatives, and, metal-chelated complexes, HPLC only quantified the unmodified Pg3,5dG standard-matched peak. This finding is consistent with previous studies: in purple asparagus, C3,5dG, C3G, and peonidin are the major pigments [45]; in the skin of ‘Tailihong’ jujube, Pg3,5dG and cyanidin-3-*O*-rutinoside together give the tissue a reddish-purple phenotype [9]. Notably, cultivated strawberries such as ‘Benihoppe’ primarily accumulate Pg3G, which gives them a red color [42], while the combination of C3,5dG, C3G, and Pg3,5dG results in a reddish-purple phenotype [9]. This study revealed that *FaTRAB1* specifically promotes the accumulation of C3,5dG, C3G, and Pg3,5dG in strawberry leaves, thereby imparting a reddish-purple phenotype to the tissue, providing novel insights into the molecular regulatory network of strawberry anthocyanin biosynthesis.

### 3.2. FaTRAB1directly activates *FaF3’H, FaANS, FaUFGT,* and *FaOMT* to promote anthocyanin accumulation

Structural genes encoding core enzymes (e.g., *CHS, F3’H, DFR, ANS, UFGT, OMT* etc.) are essential for the stepwise catalysis of anthocyanin biosynthesis, directly determining the formation, modification, and accumulation of the anthocyanin backbone structure, thus affecting plant coloration, antioxidant activity, and environmental adaptability. As the determinant enzyme for cyanidin biosynthesis, *F3’H* mediates the branch point between pelargonidin (non-hydroxylated) and cyanidin (3’-hydroxylated) anthocyanin pathways. *MdF3’H* helps accumulate cyanidin in *Arabidopsis* [46]. ANS catalyzes the conversion of colorless anthocyanidins into colored anthocyanins, while UFGT is responsible for the glycosylation modification of anthocyanins, jointly determining the stability and accumulation of anthocyanins, thereby affecting plant color expression [47]. OMTs methylate cyanidin glycosides, contributing to purple pigmentation in peonies [48]. The expression of anthocyanin structural genes is transcriptionally controlled by MYB, WRKY, bZIP, and ERF regulators, which collectively modulate plant coloration through pathway activation. Such as MdERF109, MdbZIP23 and PavMYB.C2 specifically regulate *UFGT* to increase anthocyanin accumulation in apple calli and sweet cherries [23,38,49], MdWRKY11 and VmMYBA1 activate *F3H, FLS*, *DFR* and *ANS* in apple and blueberries [50,51], MsERF17, MpbZIP9, and McMYB10 activates *F3’H* in Chinese flowering crabapple, apple, and crabapple [27,36,52]. In strawberries, FaBBX22, bZIP TFs FvHY5 and FaHY5 has been found to promote anthocyanin biosynthesis [15,19,28]. In this study, the expression of *FaTRAB1* in strawberries increased the transcription and expression levels of anthocyanin biosynthesis-related genes such as *FaPAL, FaC4H, Fa4CL, FaF3H, FaF3’H, FaDFR, FaANS, FaUFGT*, and *FaOMT* in strawberry leaves (S3 Fig). Y1H assays confirmed that FaTRAB1 activate anthocyanin structural genes (*FaF3’H, FaANS, FaUFGT,* and *FaOMT*) (Fig 6). In strawberry leaves, this regulatory network primarily promotes the significant accumulation of C3,5dG, C3G, and Pg3,5dG, resulting in a characteristic reddish-purple phenotype in the leaves. However, in fruits, the accumulation patterns of anthocyanins are markedly different: *FaTRAB1* expression alone only increased the level of the unstable intermediate CY, while co-expression with *FaF3’H* promoted the conversion of CY to the colored compound C3G. Overexpression of *FaF3’H* or *FaTRAB1* + *FaF3’H* resulted in the highest accumulation of Pg3G, C3G, and CY derivatives in strawberry fruits (Fig 8G–I). This phenomenon may be attributed to the pivotal role of F3’H at the metabolic branch point of the flavonoid pathway, where it regulates the conversion of dihydrokaempferol to dihydroquercetin, thereby increasing the precursor pool for cyanidin derivatives and promoting C3G and CY accumulation. Concurrently, the endogenous activities of DFR, ANS, and UFGT—which remain highly active during anthocyanin biosynthesis in ‘Benihoppe’ strawberry [42]—may be further enhanced by *F3’H* overexpression, acting as an upstream regulatory gene to elevate Pg3G levels in fruits. The overexpression of *FaTRAB1* + *FaF3’H* may activate the *F3’H* promoter and synergize with the MBW complex to enhance the expression of structural genes, thereby promoting the accumulation of pelargonidin- and cyanidin-type derivatives. Notably, although *FaOMT* is involved in anthocyanin modification in leaves, overexpression of *FaOMT* in fruits did not detect peonidin, which can be attributed to two primary reasons. Firstly, the inherently low activity of FaF3’H in ‘Benihoppe’ strawberry severely limits the production of critical substrates (C3G and C3,5dG) necessary for peonidin biosynthesis [53]. Secondly, as established in previous studies, pelargonidin-3-glucoside (Pg3G) dominates as the major anthocyanin in this cultivar [54], and its overwhelming abundance likely obscures the detection of any trace amounts of peonidin that might be produced. These findings demonstrate that FaTRAB1, through organ-specific regulation, controls the synthesis pathways of cyanidin and peonidin in leaves, while in fruits, it primarily participates in regulating cyanidin metabolism in collaboration with *FaF3’H,* leading to differentiated color patterns in different organs.

### 3.3. FaTRAB1 forms a new complex with FaMYB10 and FaTTG1 to participate in anthocyanin accumulation

**Fig 10:**
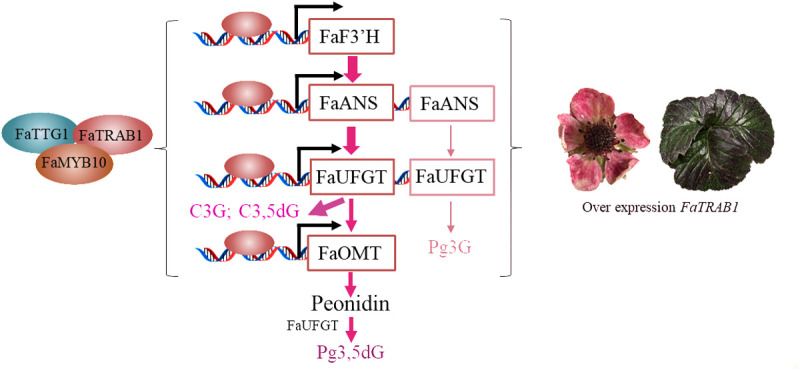
FaTRAB1 participated in regulating anthocyanin structural genes to promote anthocyanin accumulation in ‘Ruegen’ strawberry leaves. Cyanidin-3,5-*O*-diglucoside (C3,5dG), peonidin-3,5-*O*-diglucoside (Pg3,5dG), cyanidin-3-*O*-glucoside (C3G), pelargonidin-3-*O*-glucoside (Pg3G), flavonoid 3’-hydroxylase (F3’H), anthocyanidin synthase (ANS), UDP-glucose: flavonoid 3-O-glucosyltransferase (UFGT), O-Methyltransferase (OMT)(I), MYB transcription factor 10 (MYB10), transparent testa glabra 1 (TTG1).

The MBW complex, formed by MYB-bHLH-WD40, plays a central role in regulating anthocyanin biosynthesis in plants. Studies have shown that various regulatory factors influence anthocyanin synthesis by affecting the formation of the MBW complex: for example, the pear PpHY5-PpBBX18 module activates PpMYB10 to promote anthocyanin accumulation [55]. However, PpBBX21 interferes with the transcriptional activation activity of PpHY5-PpBBX18 by forming a PpBBX21-PpHY5/PpBBX18 repressive complex [55]. Similarly, PpMYB18 [56], MdbHLH162 [57], and PpMYB140 [58] inhibit MBW complex formation and anthocyanin accumulation by competitively binding to bHLH sites. We found that FaTRAB1 interacts with FaMYB10 and FaTTG1 but does not bind to FabHLH3 (Fig 7), suggesting that it may competitively interfere with the formation of the FaMYB10-FaTTG1-FabHLH3 complex. In fruits, FaTRAB1 slightly inhibits the anthocyanin accumulation of Pg3G and CY mediated by FaMYB10-FaTTG1 (Fig 9), possibly because FaTRAB1 activates FaF3’H, diverting the anthocyanin substrate dihydrokaempferol and significantly reducing the Pg3G content (Fig 10). In leaves, FaTRAB1 promotes anthocyanin synthesis through three unique mechanisms: 1) upregulating the expression of FaMYB10, FaTTG1, and FabHLH3 (S3 J–L Fig) FaTRAB1 forms a new complex with FaMYB10-FaTTG1; FaTRAB1 upregulated anthocyanin structural genes (*FaF3’H, FaANS, FaUFGT,* and *FaOMT*). This tissue-specific regulatory pattern is similar to the mechanism of AcMYB96 in onions, which promotes anthocyanin synthesis by activating *AcCHS1, AcANS*, and *AcUFGT*, without interacting with bHLH [59]. In wild strawberries, the FvMYB10L-FvTT8 complex only activates *FvDFR2*, causing the petioles to accumulate cyanidin and peonidin, resulting in a purplish-red color; in fruits, however, both *FvDFR1* and *FvDFR2* need to be co-expressed to synthesize anthocyanins [16]. These findings expand our understanding of the organ-specific coloration mechanisms in plants. Additionally, protein interactions can enhance transcription factors’ regulation of anthocyanin genes: apple MdMYB308L-MdbHLH33 promotes *MdDFR* expression [10], pear PpMYB10-PpbHLH64 activates *PpUFGT* [13], and the grape VvMYBA2r-VvMYCA1-VvWDR1 complex regulates multiple anthocyanin genes [14]. These discoveries provide new insights into how FaTRAB1 efficiently promotes anthocyanin accumulation in strawberry leaves (but not fruits)—potentially by forming specific protein complexes to achieve tissue-specific regulation. In fruits, we may reduce Pg3G content and increase the accumulation of other anthocyanins by using a strategy that diverts anthocyanin substrate synthesis, thereby altering fruit color.

## 4. Conclusion

This study reveals the multifunctional role of FaTRAB1 in regulating the accumulation of anthocyanins, chlorophyll, and lignin in strawberry leaves, with a focused investigation into the anthocyanin accumulation molecular mechanism. In ‘Ruegen’ strawberry leaves, FaTRAB1 forms a functional complex with FaMYB10 and FaTTG1, collaboratively activating anthocyanin structural genes—*FaF3’H, FaANS, FaUFGT*, and *FaOMT*—to drive tissue-specific accumulation of cyanidin-type compounds (mainly C3,5dG and C3G, with Pg3,5dG as a secondary compound), with the least accumulation of Pg3G, ultimately resulting in a purplish-red phenotype in the leaves (Fig 10). In contrast, strawberry fruits are characterized by the accumulation of Pg3G. The co-expression of *FaTRAB1 + FaMYB10/FaTTG1* may mildly inhibit the effects of *FaMYB10* or *FaTTG1* in promoting Pg3G and CY anthocyanin accumulation in strawberry fruits by interfering with the FaMYB10-FaTTG1-FabHLH3 complex. FaTRAB1 is more likely to specifically regulate the activity of *FaF3’H*, leading to the diversion of the anthocyanin substrate dihydrokaempferol and promoting the accumulation of C3G in the fruit. This study revealed the molecular regulatory mechanism of anthocyanin accumulation in strawberry leaves and explains how *FaTRAB1* regulates the differential accumulation of anthocyanins in strawberry leaves and fruits through tissue-specific regulation, providing new insights for developing strawberry cultivars with different anthocyanin types and contents.

## 5. Materials and methods

### 5.1. Plant materials

For transient overexpression and stable transformation, we selected two strawberry genotypes: the cultivated variety *Fragaria*× *ananassa* ‘Benihoppe’ and the wild-type *Fragaria* vesca ‘Ruegen’. Both were grown in a controlled plastic greenhouse at Sichuan Agricultural University (from May 2023 to May 2024). Fruits of ‘Benihoppe’ at the de-greening stage were harvested for *Agrobacterium*-injection [60], ten fruits were one biological replicate, including three biological replicates. 5 wild-type seedlings and 5 lines of stably overexpression FaTRAB1 plants were removed from their containers, and then transplanted into a sterilized substrate. Subsequently, plants were cultivated in a greenhouse (70–80% humidity, 23 ± 2^o^C).

### 5.2. RNA extraction, cDNA synthesis and PCR amplification

Strawberry total RNA was isolated with a plant RNA extraction kit (TianGen, China), followed by first-strand cDNA synthesis using RevertAid H Minus Reverse Transcriptase (Thermo Fisher, USA). PCR amplification (20 μL reaction volume, 34 cycles) was conducted with primers provided in Table C in S1 Text.

### 5.3. Identification of transgenic strawberry lines

The overexpression vectors were introduced into ‘Ruegen’ strawberry via *Agrobacterium*-mediated transformation. Transgenic plants were selected on hygromycin-containing medium, followed by rooting on 1/2 MS medium for 2 months. Primary screening was performed using GUS staining of leaves, with positive transformants subsequently verified through RT-qPCR analysis of *FaTRAB1* expression levels (primers in Table C in S1 Text).

### 5.4. Identification of *FaTRAB1* gene

We searched for homologous protein sequences of FaTRAB1 (FxaC7g04340) in other Rosaceae species (diploid strawberry, rose, peach, pear) through the GDR database (https://www.rosaceae.org/), as well as in Arabidopsis and tomato using the TAIR database (https://www.arabidopsis.org/) and the Phytozome database (https://phytozome-next.jgi.doe.gov/). A total of 15 sequences were obtained. The resulting protein sequences were submitted to three major databases for domain identification: NCBI CDD (https://www.ncbi.nlm.nih.gov/Structure/bwrpsb/bwrpsb.cgi), Pfam (http://pfam.xfam.org/search#tabview=tab1), and SMART (http://smart.embl.de/#), and those containing the target domain b-ZIP (bZIP-plant-BZIP46) were retained. We then aligned the FaTRAB1 sequence with the orthologous sequences from other plant species using Mega 11 and constructed a phylogenetic tree using the neighbor-joining method. The motif elements and domains were visualized using TBtools.

### 5.5. Subcellular localization analysis

*FaTRAB1* eGFP fusion vectors were constructed. The empty vector 35s::eGFP was mixed with RFP and RPP3A-mCherry in equal proportions for injection as control. Equal mixtures of RPP3A-mCherry (cytosolic marker), NLS-mCherry (nuclear marker) and eGFP fusion vector were agroinfiltrated (GV3101 strain) into N. *benthamiana* leaves. After 3 days, fluorescence was visualized by confocal microscopy (Olympus FV3000, Japan). See Table C in S1 Text for eGFP fusion vector primers.“

### 5.6. Determination of indicators of fruit quality

Total anthocyanins were quantified by pH differential method [61]. The chlorophyll content in strawberry leaves was measured using spectrophotometry. Fruit sugar, organic acid, and anthocyanin (peonidin-3,5-*O*-diglucoside, Pg3,5dG; cyanidin-3-*O*-glucoside, C3G; cyanidin-3,5-*O*-diglucoside, C3,5dG) contents were analyzed by HPLC (Agilent system). An Athena NH2-RP column was used for sugars, and an Athena C18-WP column for organic acids and anthocyanins. Quantification was performed using corresponding external standards (Sigma, USA), with three biological replicates for each analysis.

### 5.7. Transmission electron microscopy analysis of strawberry leaf chloroplasts

Transmission electron microscopy was performed according to the method of Huang et al [62]. Briefly, strawberry leaves were cut into rectangular strips of approximately 0.5 mm × 2 mm and fixed in a solution of 4% glutaraldehyde and 3% paraformaldehyde. Approximately 10 pieces of each sample were placed into 2 mL centrifuge tubes and fixed at room temperature for 4 hours, followed by overnight fixation at 4^o^C. After dehydration through an alcohol gradient, infiltration with spur-resin/acetone, spur-fixation, and embedding in plastic, five longitudinal ultrathin sections were prepared for each sample. Three randomly selected ultrathin sections were placed on 100-mesh copper grids. Chloroplast structures were observed under a transmission electron microscope (TECNAI 12, Philips, The Netherlands).

### 5.8. LC-MS/MS analysis

Anthocyanins were quantified using a UPLC-ESI-MS/MS system (AB Sciex QTRAP 6500) via scheduled multiple reaction monitoring (MRM). Data were acquired with Analyst 1.6.3 (Sciex) and processed using Multiquant 3.0.3 (Sciex) for metabolite quantification. Metabolite profiling was performed by MetWare (Wuhan, China). Significantly regulated anthocyanins were identified with a threshold of |Log₂FC| ≥ 1 (fold change ≥ 2 or ≤ 0.5) between OE-FaTRAB1 and WT groups (three biological replicates per group).

### 5.9. Transcriptome analysis

Total RNA was extracted from leaves of two groups. RNA-seq library construction was performed following the standard protocol of the Illumina Next Ultra RNA Library Prep Kit (NEB, USA). Four libraries were clustered and sequenced (150 bp, paired-end) by NovoGene (Beijing, China) using the HiSeq-2500 platform. FASTQ reads generated from CASAVA base calling were filtered for low-quality bases (Q < 20) and adapter sequences using Trim Galore (v0.6.6). The cleaned reads were aligned to the strawberry genome (*Fragaria vesca* Whole Genome v4.0.a1 Assembly & Annotation). Transcripts with significant changes (log_2_ fold change > 0.5 or <-0.5) and an adjusted *p*-value ≤ 0.05 were considered differentially ex*p*ressed. All experiments were performed with three biological replicates.

### 5.10. Y1H, LUC and EMSA analysis

#### 5.10.1. Yeast one-hybrid (Y1H) assays.

We first predicted the potential binding cis-elements between *FaTRAB1* and *FaF3’H*, *FaANS*, *FaUFGT*, and *FaOMT* using JASPAR (https://jaspar.elixir.no/).The *cis*-elements of *FaF3’H* (AACAATACGTGAT), *FaANS* (TGACGTGTCA), *FaUFGT* (TGACCCATGGCGACGAAAC) and *FaOMT* (ACGTGTA) each with two or three tandem repeats, were cloned into pAbAi to form the bait vector, while *FaTRAB1* full-length cDNA was inserted into pGADT7. The bait vector was transformed into Y1H yeast, followed by the pGADT7- *FaTRAB1* prey vector. Transformants were validated using 1 × , 10 × , and 100 × dilutions on/-Leu/AbA plates. Y1H primers are listed in Table C in S1 Text.

#### 5.10.2. Dual-luciferase assay.

The *FaTRAB1* coding sequence was cloned into pCambia1301 (effector), while the 2-kb FaF3’H, FaANS, FaUFGT and FaOMT promoters were inserted into pGreenII 0800-LUC (reporter) (primers in Table C in S1 Text). These constructs were transformed into *Agrobacterium tumefaciens* GV3101 and co-infiltrated into tobacco leaves (1:9 ratio). After 3 days, dual-luciferase assays (Yeasen, Shanghai) determined LUC/REN ratios. For imaging, leaves were sprayed with 1 mM D-luciferin (Yeasen, Shanghai), and luminescence was captured after 10 min using a Lumazone Pylon 2048B system (USA).

#### 5.10.3. Electrophoresis mobility shift assay (EMSA) assays.

The full-length cDNAs sequence of *FaTRAB1* was cloned into pMAL-c5x (MBP tag) and expressed it in *E. coli* BL21. MBP- *FaTRAB1* was purified from the supernatant (Thermo Fisher, Waltham, MA, USA). The DNA fragments of the promoter regions with corresponding binding elements for the downstream genes were labeled with biotin and used as probes (Biorun, Wuhan, China). EMSA was conducted with the LightShift Chemiluminescent EMSA Kit (Thermo Fisher, USA), using primers and probes listed in Table C and D in S1 Text, respectively.

### 5.11. Y2H, LCI, BiFC and CoIP assays

#### 5.11.1. Yeast two-hybrid (Y2H) assays.

The cDNAs of *FaMYB10/FaTTG1/FabHLH3* and *FaTRAB1* were fused to GAL4 activation (pGADT7) and binding (pGBKT7) domains, respectively. Following co-transformation into Y2HGold yeast strain, transformants were selected on SD/-Leu/-Trp, and SD/-Leu/-Trp/-Ade/-His + X-α-gal (0.2 mg/mL) for interaction screening. The appearance of blue colonies within 3–5 days at 28^o^C confirmed protein-protein interaction. Primers are documented in Table C in S1 Text.

#### 5.11.2. Luciferase complementation imaging (LCI) assays.

The full-length cDNAs of *FaTRAB1* and *FaMYB10/FaTTG1/FabHLH3* were cloned into p1300-nLuc and p1300-cLuc vectors, respectively (primers in Table C in S1 Text). *Agrobacterium* mixtures containing nLuc-FaTRAB1 and FaMYB10/FaTTG1/FabHLH3 (1:1 ratio) were infiltrated into tobacco leaves, with three negative controls (empty vector combinations). The luminescence was detected three day post infiltration using 1 mM D-luciferin substrate. Images were acquired after 10 min incubation (Lumazone Pylon 2048B, Princeton, USA).

#### 5.11.3. Bimolecular fluorescence complementation (BiFC) assays.

The full-length cDNAs of *FaTRAB1* and *FaMYB10/FaTTG1/FabHLH3* were cloned into pXY104-cEYFP and pXY103-nEYFP vectors, respectively (primers in Table C in S1 Text). *Agrobacterium* mixtures containing FaMYB10/FaTTG1/FabHLH3-nEYFP and FaTRAB1-cEYFP (1:1 ratio) were co-infiltrated into tobacco leaves, with two non-interacting controls (cEYFP +FaMYB10/FaTTG1/FabHLH3-nEYFP and FaTRAB1-cEYFP + nEYFP). Fluorescence complementation was observed 3 days post-infiltration using confocal microscopy (Olympus FV3000, Japan).

#### 5.11.4. Co-inmunoprecipitation (CoIP) assays.

Tobacco leaves were co-infiltrated with *Agrobacterium* tumefaciens GV3101 strains carrying either FaTRAB1-GFP or combinations of FaMYB10-MYC/FaTTG1-MYC constructs. Total proteins were extracted 48 hours post-infiltration and subjected to Co-IP using anti-GFP magnetic beads (BIORUN BIOSCIENCES CO., LTD., Cat# HY-K0207) according to the manufacturer’s protocol.

### 5.12. Transient overexpression of genes in strawberry fruit

We cloned the full-length coding sequences of *FaTRAB1*, *FaF3’H, FaANS, FaUFGT, FaOMT, FaMYB10* and *FaTTG1* (primers in Table C in S1 Text) into the pCAMBIA1301 expression vector, with empty vector serving as control. These constructs were introduced into de-greening stage strawberry fruits through *Agrobacterium*-mediated transformation. Five days post-infiltration, we assessed fruit ripening parameters in three biological replicates, each consisting of ten fruits per treatment.

### 5.13. RT-qPCR assays

RT-qPCR was performed in 20 μL reaction volumes with 34 cycles, using FaActin2 (LOC101313255) as the internal reference. Relative transcript levels were calculated via 2^−ΔΔCt^ analysis with triplicate biological replicates. Primer pairs are suppled in Table E in S1 Text.

### 5.14. Statistical analysis

Statistical analysis was performed using IBM SPSS Statistics (v25.0), with data presented as mean ± SD. Significant differences were determined by independent samples t-test, and with * indicating **p* *≤ 0.05 and ** indicating *p* ≤ 0.01. Significant variations across samples are also shown by different lowercase letters (LSD’s multiple range test).

## Supporting information

S1 Fig Identification of transgenic strawberry lines. Gus staining results of overexpressed lines (A). RT-qPCR of FaTRAB1 in the transgenic lines (B).
(TIF)

S2 Fig Statistical analysis of differentially expressed genes in the transcriptome (A) and KEGG enrichment analysis (B). (TIF)

S3 Fig Quantification of genes related to the anthocyanin metabolism pathway. Phenylalanine ammonia-lyase (PAL) (A), cinnamate 4-hydroxylase (C4H) (B), 4-Coumarate-CoA ligase (4CL) (C), flavanone 3-hydroxylase (F3H) (D), flavonoid 3’-hydroxylase (F3’H) (E), dihydroflavonol 4-reductase (DFR) (F), anthocyanidin synthase (ANS) (G), UDP-glucose: flavonoid 3-O-glucosyltransferase (UFGT) (H), O-Methyltransferase (OMT) (I), MYB transcription factor 10 (MYB10) (J), transparent testa glabra 1 (TTG1) (K), basic Helix-Loop-Helix 3 (bHLH3) (L). YL: young leaf; ML: mature leaf; OL: old leaf. Statistical significance is indicated as follows: *p ≤ 0.05, **p ≤ 0.01.
(TIF)

S1 Text Table A in S1 Text. Differential expression metabolites in strawberry leaves of overexpressing FaTRAB1.Table B in S1 Text. Differentially expressed genes (DEGs) in the lignin and anthocyanin biosynthetic pathways. Table C in S1 Text. Primers used for plasmid construction in this study. Table D in S1 Text. Probe and mutant probe information. Table E in S1 Text. Primers used for gene expression analysis in this study.
(DOCX)
