# Supplementary figures and images for: FaTRAB1, a bZIP transcription factor, enhances anthocyanin biosynthesis in strawberry leaves via tissue-specific regulation
Source: PLoS Genet. 2025 Sep 24;21(9):e1011888. doi: 10.1371/journal.pgen.1011888 (PMC12494280; doi:10.1371/journal.pgen.1011888)

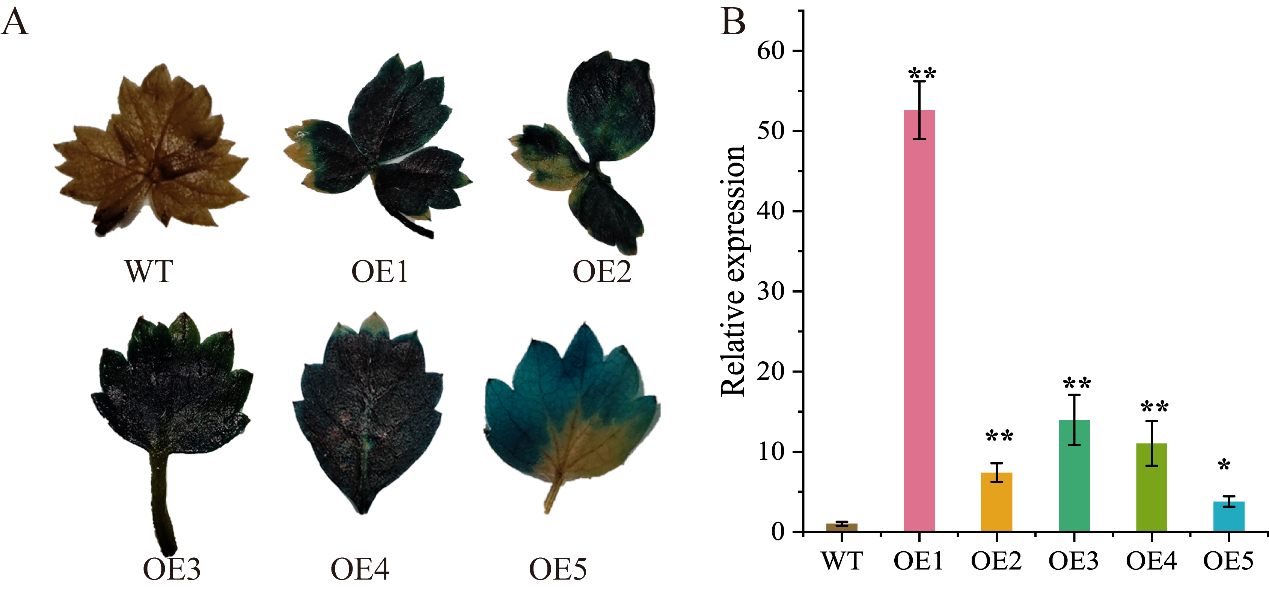

Supplement: S1 Fig — Gus staining results of overexpressed lines (A). RT-qPCR of FaTRAB1 in the transgenic lines (B). (TIF) [file pgen.1011888.s001.tif]

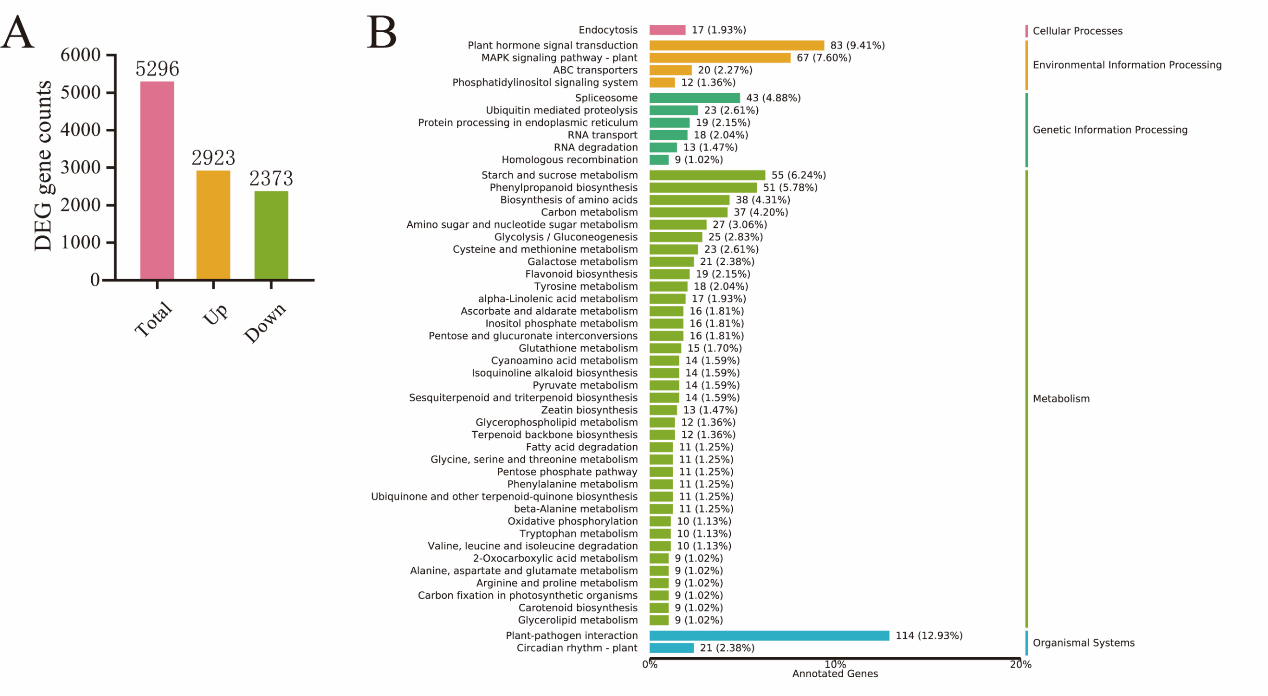

Supplement: S2 Fig — (TIF) [file pgen.1011888.s002.tif]

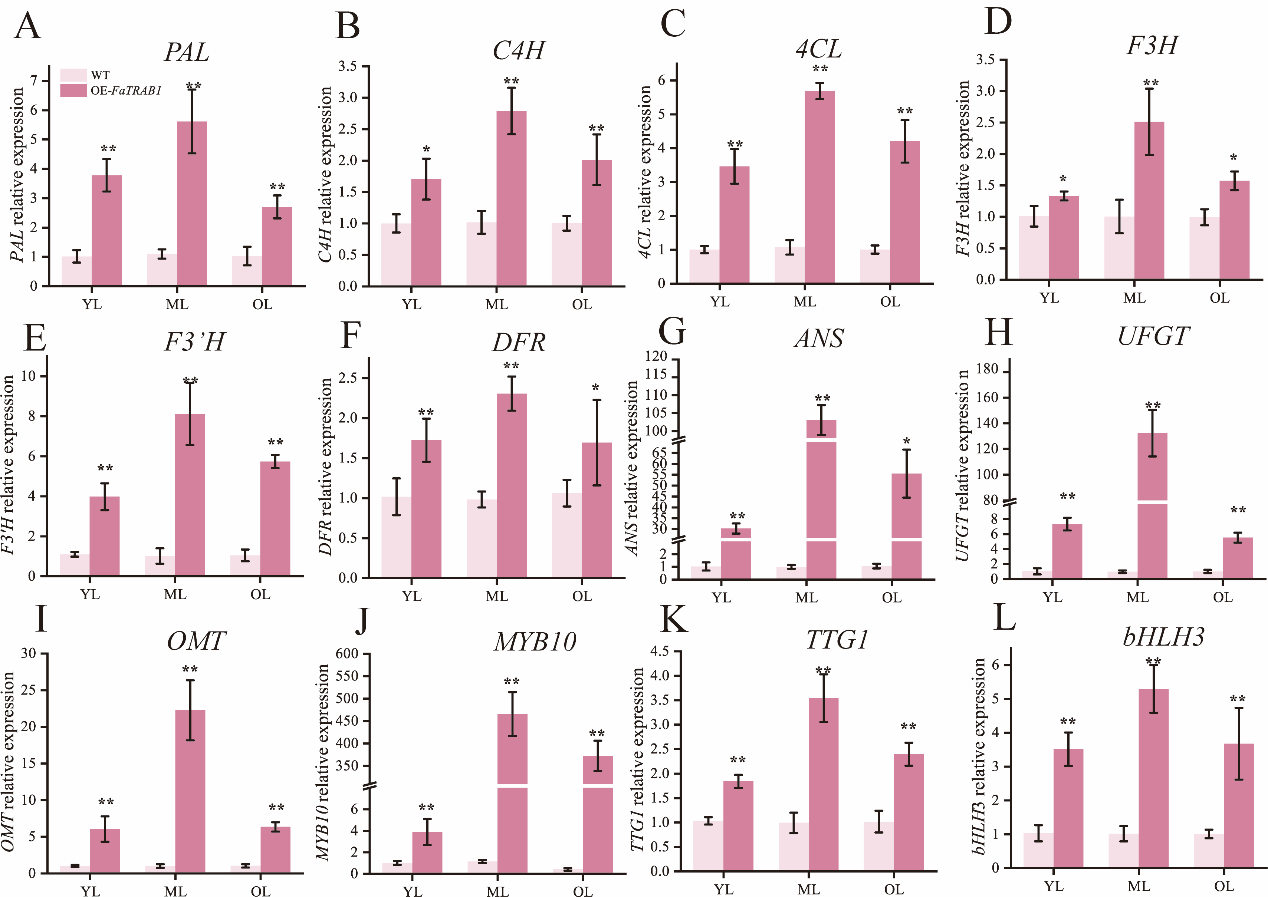

Supplement: S3 Fig — Phenylalanine ammonia-lyase (PAL) (A), cinnamate 4-hydroxylase (C4H) (B), 4-Coumarate-CoA ligase (4CL) (C), flavanone 3-hydroxylase (F3H) (D), flavonoid 3’-hydroxylase (F3’H) (E), dihydroflavonol 4-reductase (DFR) (F), anthocyanidin synthase (ANS) (G), UDP-glucose: flavonoid 3-O-glucosyltransferase (UFGT) (H), O-Methyltransferase (OMT) (I), MYB transcription factor 10 (MYB10) (J), transparent testa glabra 1 (TTG1) (K), basic Helix-Loop-Helix 3 (bHLH3) (L). YL: young leaf; ML: mature leaf; OL: old leaf. Statistical significance is indicated as follows: *p ≤ 0.05, **p ≤ 0.01. (TIF) [file pgen.1011888.s003.tif]
