# Supplementary material for: FaTRAB1, a bZIP transcription factor, enhances anthocyanin biosynthesis in strawberry leaves via tissue-specific regulation
Source: PLoS Genet. 2025 Sep 24;21(9):e1011888. doi: 10.1371/journal.pgen.1011888 (PMC12494280; doi:10.1371/journal.pgen.1011888)
Supplement: S1 Text — Table A in S1 Text. Differential expression metabolites in strawberry leaves of overexpressing FaTRAB1.Table B in S1 Text. Differentially expressed genes (DEGs) in the lignin and anthocyanin biosynthetic pathways. Table C in S1 Text. Primers used for plasmid construction in this study. Table D in S1 Text. Probe and mutant probe information. Table E in S1 Text. Primers used for gene expression analysis in this study. (DOCX) [file pgen.1011888.s004.docx]

Supporting information

Table A in S1 Text. Differential expression metabolites in strawberry leaves of overexpressing FaTRAB1

Table B in S1 Text. Differentially expressed genes (DEGs) in the lignin and anthocyanin biosynthetic pathways

Table C in S1 Text. Primers used for plasmid construction in this study.

Table D in S1 Text. Probe and mutant probe information

Table E in S1 Text. Primers used for gene expression analysis in this study

Table A in S1 Text. Differential expression metabolites in strawberry leaves of overexpressing *FaTRAB1*

Table B in S1 Text. Differentially expressed genes (DEGs) in the lignin and anthocyanin biosynthetic pathways

Table C in S1 Text. Primers used for plasmid construction in this study.

Table D in S1 Text. Probe and mutant probe information

Table E in S1 Text. Primers used for gene expression analysis in this study

Table A. Differential expression metabolites in strawberry leaves of overexpressing *FaTRAB1*

| Compounds | WT | OE-FaTRAB1 | Categorization |
| --- | --- | --- | --- |
| Cyanidin-3-O-glucoside | 76.08 | 751.21 | Cyanidin |
| Cyanidin-3,5-O-diglucoside | 123.49 | 620.78 |  |
| Cyanidin-3-O-xyloside | 0.11 | 4.02 |  |
| Cyanidin-3-O-rutinoside | 0.14 | 0.48 |  |
| Cyanidin-3-malonyl-glucosyl-glucoside | 0.11 | 0.04 |  |
| Cyanidin-3-O-sambubioside | 0.69 | 10.56 |  |
| Cyanidin-3-O-(6-O-p-coumaroyl)-glucoside | 0.25 | 1.75 |  |
| Cyanidin-3-O-arabinoside | 0.02 | 0.19 |  |
| Cyanidin-3-[6''-(Galloy)rhamnoside] | 0.00 | 0.03 |  |
| Cyanidin-caffeoyl-glucoside-glucoside | 0.00 | 0.03 |  |
| Cyanidin-3-O-sambubioside-5-O-glucoside | 0.14 | 0.00 |  |
| Cyanidin-3-xylosyl-galactoside | 0.52 | 0.00 |  |
| Cyanidin3-O-(6''-O-feruloyl)glucoside-5-O-glucoside | 0.02 | 0.00 |  |
| Cyanidin-3-pentoside | 0.02 | 0.00 |  |
| Cyanidin-3-xylosyl-glucoside | 0.06 | 0.00 |  |
| Cyanidin-3-O-5-O-(6-O-coumaroyl)-diglucoside | 0.01 | 0.00 |  |
| Cyanidin-3-O-(hydroxybenzoyl)glucoside | 0.01 | 0.00 |  |
| Pelargonidin-3-O-sophoroside | 0.02 | 0.13 | Pelargonidin |
| Pelargonidin-3-O-glucoside | 13.60 | 244.55 |  |
| Pelargonidin-3,5-O-diglucoside | 3.30 | 20.48 |  |
| Pelargonidin-3,5-O-Digalactoside | 0.05 | 1.15 |  |
| Pelargonidin-3-O-xyloside | 0.01 | 0.85 |  |
| Pelargonidin-3-O-(6''-O-acetyl)glucoside | 0.00 | 0.06 |  |
| Pelargonidin | 0.00 | 0.02 |  |
| Pelargonidin-3-O-arabinoside | 0.00 | 0.22 |  |
| Pelargonidin-3-O-rutinoside | 0.30 | 0.00 |  |
| Peonidin-3-O-(6-O-p-coumaroyl)-galactoside | 0.01 | 0.02 | Peonidin |
| Peonidin-3,5-O-diglucoside | 300.46 | 1026.81 |  |
| Peonidin-3-O-glucoside | 13.10 | 89.16 |  |
| Peonidin-3-O-sambubioside | 0.15 | 1.54 |  |
| Peonidin-3-O-(caffeoyl)rhamnoside | 0.07 | 0.25 |  |
| Peonidin-3-O-(6-O-p-coumaroyl)-glucoside | 0.00 | 0.02 |  |
| Peonidin-3-O-5-O-(6-O-coumaroyl)-diglucoside | 0.00 | 0.05 |  |
| Peonidin-3-O-galactoside | 0.45 | 0.00 |  |
| Delphinidin-3-O-rutinoside | 0.17 | 0.07 | Delphinidin |
| Delphinidin-3-O-glucoside-5-O-galactoside | 0.02 | 0.09 |  |
| Delphinidin-3-O-(6''-O-coumaroyl)rhamnoside-5-O-glucoside | 0.01 | 0.01 |  |
| Delphinidin-3-O-(6''-O-acetyl)galactoside | 0.14 | 0.56 |  |
| Delphinidin-caffeoyl-rutinoside | 0.01 | 0.00 |  |
| Quercetin-3-O-glucoside | 46.66 | 102.47 | Quercetin |
| Malvidin-3,5-O-diglucoside | 0.13 | 0.06 | Malvidin |
| Petunidin-3-O-sambubioside | 0.00 | 0.00 | Petunidin |

Table B. Differentially expressed genes (DEGs) in the lignin and anthocyanin biosynthetic pathways

| Gene name | Gene ID |
| --- | --- |
| *CCR*  *COMT*  *HCT*  *CAD*  *CHS*  *F3’H*  *F3’5’H*  *FLS*  *F3H*  *ANS*  *ANR*  *3,5GT*  *3GT*  *UFGT*  *UGT* | FvH4_5g26090, FvH4_5g26030, FvH4_5g22420  FvH4_7g32990  FvH4_6g28410  FvH4_2g38060, FvH4_2g05150  FvH4_2g02110  FvH4_5g00510  FvH4_5g00570  FvH4_2g26440  FvH4_7g24550, FvH4_1g11810  FvH4_5g01170  FvH4_5g04260, FvH4_5g33950, FvH4_7g16120  FvH4_5g31920, FvH4_7g30540  FvH4_2g40150, FvH4_7g33840, FvH4_6g39310, FvH4_1g13990  FvH4_2g05600, FvH4_7g23180  FvH4_1g00410, FvH4_1g00390, FvH4_2g17430, FvH4_2g03300  FvH4_4g29060, FvH4_2g36640, FvH4_1g30110, FvH4_6g50530 |

Table C. Primers used for plasmid construction in this study.

| Gene | Primers |
| --- | --- |
| OE-*FaTRAB1*-F | CGCGGTGGCGGCCGCTCTAGAGTAAATGGTGGGAAGCTAGGTGG |
| OE-*FaTRAB1-R*  OE-*FaF3’H-F*  OE-*FaF3’H-R*  OE-*FaANS-F*  OE-*FaANS-R*  OE-*FaUFGT-F*  OE-*FaUFGT-R*  OE-*FaOMT-F*  OE-*FaOMT-R*  OE-*FaMYB10-F*  OE-*FaMYB10-R*  OE-*FaTTG1-F*  OE-*FaTTG1-R*  pGADT7- *FaTRAB1-F*  pGADT7- *FaTRAB1-R*  pAbAi- *FaF3’H -F*  pAbAi- *FaF3’H-R*  pAbAi-*FaANS-F*  pAbAi-*FaANS* *-R*  pAbAi-*FaUFGT-F*  pAbAi-*FaUFGT-R*  pAbAi-*FaOMT-F*  pAbAi-*FaOMT-R*  pGreen-*FaF3’H-F*  pGreen-*FaF3’H-R*  pGreen-*FaANS-F*  pGreen-*FaANS-R*  pGreen-*FaUFGT-F*  pGreen-*FaUFGT-R*  pGreen-*FaOMT-F*  pGreen-*FaOMT-R*  GFP*-FaTRAB1-F*  GFP*-FaTRAB1-R*  *pGBKT7-FaTRAB1-F*  *pGBKT7-FaTRAB1-R*  pGADT7-*FaMYB10*-*F*  pGADT7-*FaMYB10*-*R*  pGADT7-*FaTTG1*-*F*  pGADT7-*FaTTG1-R*  pGADT7-*FabHLH3*-*F*  pGADT7-*FabHLH3*-*R*  nLuc-*FaTRAB1*-*F*  nLuc-*FaTRAB1-R*  cLuc-*FaMYB10-F*  cLuc-*FaMYB10-R*  cLuc-*FaTTG1-F*  cLuc-*FaTTG1-R*  cLuc-*FabHLH3-F*  cLuc-*FabHLH3-R*  cEYFP- *FaTRAB1-F*  cEYFP- *FaTRAB1-R*  nEYFP- *FaMYB10-F*  nEYFP- *FaMYB10-R*  nEYFP- *FaTTG1-F*  nEYFP- *FaTTG1-R*  nEYFP- *FabHLH3-F*  nEYFP- *FabHLH3-R*  pMAL-*FaTRAB1-F*  pMAL-*FaTRAB1-R* | GATCTGCAGCCCGGGGGATCCCAATGTTCGTCTCAAGCACTG  CGCGGTGGCGGCCGCTCTAGACAACCATGTTTCTCATAGCAG  TCGTCCTTGTAGTCAGATCTGATTATGAAGTTTTATAAGCATGTGG  CGCGGTGGCGGCCGCTCTAGAATGGTGACTGCTGCATCCG  TCGTCCTTGTAGTCAGATCTCTAATTAGTTGAGATGAGAGCAGCT  CGCGGTGGCGGCCGCTCTAGAGAAATGGCACCAGTATCAAACC  TCGTCCTTGTAGTCAGATCTGGTTGTGGTCATTTCCAACAATG  CGCGGTGGCGGCCGCTCTAGAGAACATGCTTCTCAAGCTGATC  TCGTCCTTGTAGTCAGATCTATGCCTTTCAATCCTCCAAAC  CGCGGTGGCGGCCGCTCTAGAATGGGGGGTTTCGGTGTGAG  TCGTCCTTGTAGTCAGATCTTCACACGTAGGAGATGTTGACTAGAT  CGCGGTGGCGGCCGCTCTAGACTCCGTCACCTACGACTCTC  TCGTCCTTGTAGTCAGATCTGCAATGGCAATCCAATCCG  GGAGGCCAGTGAATTCGTAAATGGTGGGAAGCTAGGTGG  CGAGCTCGATGGATCC CAATGTTCGTCTCAAGCACTG  AACAATACGTGAT AACAATACGTGAT AACAATACGTGAT  tcgaATCACGTATTGTTATCACGTATTGTTATCACGTATTGTTgtac  TGACGTGTCATGACGTGTCATGACGTGTCA  tcga TGACACGTCATGACACGTCATGACACGTCA gtac  TGACCCATGGCGACGAAACTGACCCATGGCGACGAAAC  tcga GTTTCGTCGCCATGGGTCAGTTTCGTCGCCATGGGTCA gtac  ACGTGTAACGTGTAACGTGTA  tcgaTACACGTTACACGTTACACGT gtac  TCGACGGTATCGATAAGCTT GGTGTAAATCTGCATCATACGTC  GCTCTAGAACTAGTGGATCC GGATTTGCCGGAGAAAAGAAG  TCGACGGTATCGATAAGCTT CTGTGTAATGATCTGCGCTAG  GCTCTAGAACTAGTGGATCC GTTCAGACGTTTGGAGTATTTG  TCGACGGTATCGATAAGCTT GCAAACACCAAGATGACACTGT  GCTCTAGAACTAGTGGATCC CTAGCTAGTGATCAAGCTACGT  TCGACGGTATCGATAAGCTT CCACACTGATTGAGACTGGC  GCTCTAGAACTAGTGGATCC TGCGGTTTGACCCGGATG  GTGGTGATAGGGCCCGGATCCGTAAATGGTGGGAAGCTAGGTGG GTGATTTTTGCGGACTCTAGA CAATGTTCGTCTCAAGCACTG  CATGGAGGCCGAATTC GTAAATGGTGGGAAGCTAGGTGG  GCCGCTGCAGGTCGAC CAATGTTCGTCTCAAGCACTG  GGAGGCCAGTGAATTCATGGGGGGTTTCGGTGTGAG  CGAGCTCGATGGATCCTCACACGTAGGAGATGTTGACTAGAT  GGAGGCCAGTGAATTCCCGATGGAGAATTCGACCCT  CGAGCTCGATGGATCCTCAAACCTTCAAGAGCTGCAT  GGAGGCCAGTGAATTCCTACTTCCGGAAATGGCGAC  CGAGCTCGATGGATCCGCCCTCTTCACTTCTGTAATGG  GGGGACGAGCTCGGTACC GTAAATGGTGGGAAGCTAGGTGG  GTACGAGATCTGGTCGAC CAATGTTCGTCTCAAGCACTG  GCGTCCCGGGGCGGTACCATGGGGGGTTTCGGTGTGAG  AAAGCTCTGCAGGTCGACTCACACGTAGGAGATGTTGACTAGAT  GCGTCCCGGGGCGGTACCCCGATGGAGAATTCGACCCT  AAAGCTCTGCAGGTCGACTCAAACCTTCAAGAGCTGCAT  GCGTCCCGGGGCGGTACCCTACTTCCGGAAATGGCGAC  AAAGCTCTGCAGGTCGACGCCCTCTTCACTTCTGTAATGG  GGTACCCGGGGATCC GTAAATGGTGGGAAGCTAGGTGG  GCCACCGCCGTCGAC CAATGTTCGTCTCAAGCACTG  GGTACCCGGGGATCCATGGGGGGTTTCGGTGTGAG  CATACCGCCGTCGACTCACACGTAGGAGATGTTGACTAGAT  GGTACCCGGGGATCCCCGATGGAGAATTCGACCCT  CATACCGCCGTCGACTCAAACCTTCAAGAGCTGCAT  GGTACCCGGGGATCCCTACTTCCGGAAATGGCGAC  CATACCGCCGTCGACGCCCTCTTCACTTCTGTAATGG  AAGCTTCAAATAAAAC GTAAATGGTGGGAAGCTAGGTGG  AGTACTCAACCAAGTC CAATGTTCGTCTCAAGCACTG |

| Genes | Probe/ mutant probe |
| --- | --- |
| *FaF3’H*  *FaANS*  *FaUFGT*  *FaOMT* | 5’biotin-AACAATACGTGAT/5’biotin-TTTTTTTTTTTTT  5’biotin-TGACGTGTCATGACGTGTCA /5’biotin-CCTTTTTTTTCCTTTTTTTT  5’biotin-TGACCCATGGCGACGAAAC/5’biotin-TTTTTTTTCCCCTTTTTTT  5’biotin- ACGTGTAACGTGTAACGTGTA /5’biotin-AAAAAAAAAAAAAAAAAAAAA |

Table D. Probe and mutant probe information

Table E. Primers used for gene expression analysis by RT-qPCR

| Genes | Primers |
| --- | --- |
| *FaActin2-*F  *FaActin2-*R  q*FaTRAB1*-F  q*FaTRAB1*-R  q*FaPAL-F*  q*FaPAL-R*  q*FaC4H-F*  q*FaC4H-R*  q*Fa4CL-F*  q*Fa4CL-R*  q*FaF3H-F*  q*FaF3H-R*  q*FaF3’H-F*  q*FaF3’H-R*  q*FaDFR-F*  q*FaDFR-R*  q*FaANS-F*  q*FaANS-R*  q*FaUFGT-F*  q*FaUFGT-R*  q*FaOMT-F*  q*FaOMT-R*  q*FaMYB10-F*  q*FaMYB10-R*  q*FaTTG1-F*  q*FaTTG1-R*  q*FabHLH3-F*  q*FabHLH3-R* | TGGGTTTGCTGGAGATGAT  CAGTTAGGAGAACTGGGTGC  GTAAATGGTGGGAAGCTAGGTGG  CCTTGAGTCTCTTCAGCACTCC  CAGAAGCAGTTGACATAC  CTTCAAGTTCTCCTCCAA  GTGAAGGAGAAGAGGATT  CATTAGTTGTGACCTGTG  GGTTATGTGGACGATGAC  ACTTGGAAGCCTTTGAAT  GGAGGTTCAAGAATGCTGAT  TCTGGAATGTCGCTATGGA  ATGGTGGTGGAGATGATG  CAAGTCAAGCCACTCAAG  TTCCACAAATCACAACAC  TTCCACCTTATGCTTCTT  GCGACACCTTAGAGATTC  CACCTTCTCCTTGTTGAC  GAAGCAATCGGCAGAGCTAG  GAAGAGTCCGCAAAGGACTC  AAGATGCTCCTCAATATCC  CTCCAGTCGTGACATATC  CAATGATGTGAAGAACTATT  TCTGAATGATTATGCTCTAT  ATCATATACGAGAGTCCT  GAATATCCAGTATCACAAC  ATGGTTCTACTTGATGTG  TTTGCTATCTACTTCATTTG |
